# Mapping Molecular Networks within *Clitoria ternatea* Linn. against LPS-Induced Neuroinflammation in Microglial Cells, with Molecular Docking and In Vivo Toxicity Assessment in Zebrafish

**DOI:** 10.3390/ph15040467

**Published:** 2022-04-12

**Authors:** Nurul Farah Adni Mat Zian, Puspanjali Swain, Siti Munirah Mohd Faudzi, Norzalina Zakaria, Wan Norhamidah Wan Ibrahim, Noraini Abu Bakar, Khozirah Shaari, Johnson Stanslas, Tae-Ik Choi, Cheol-Hee Kim

**Affiliations:** 1 Natural Medicines and Product Research Laboratory, Institute of Bioscience, Universiti Putra Malaysia, Serdang 43400, Selangor, Malaysia; nuruladni95@gmail.com (N.F.A.M.Z.); wnwi@upm.edu.my (W.N.W.I.); khozirah@upm.edu.my (K.S.); 2 Department of Biology, Chungnam National University, 99 Daehak-ro, Yuseong-gu, Daejeon 34134, Korea; puspanjaliswain20@gmail.com (P.S.); c860523@naver.com (T.-I.C.); 3 Department of Chemistry, Faculty of Science, Universiti Putra Malaysia, Serdang 43400, Selangor, Malaysia; norzalinazakaria94@gmail.com; 4 Department of Biology, Faculty of Science, Universiti Putra Malaysia, Serdang 43400, Selangor, Malaysia; norainiabubakar011@gmail.com; 5 Department of Medicine, Faculty of Medicine & Health Sciences, Universiti Putra Malaysia, Serdang 43400, Selangor, Malaysia; rcxjs@upm.edu.my

**Keywords:** butterfly pea, *Clitoria ternatea*, anti-neuroinflammation, Alzheimer’s disease, molecular networking, zebrafish toxicity

## Abstract

Clitoria ternatea Linn. (CT), or butterfly pea, is an Ayurvedic plant traditionally used as a brain tonic. Recently, it was reported to be of use in treating central nervous system (CNS) disorders, i.e., as an antistress treatment and antidepressant. In the present study, we report a detailed phytochemical profile of the ethyl acetate fraction of the flower of CT (CTF_EA) with significant neuroprotective and anti-neuroinflammatory properties in both LPS-activated BV-2 and SK-N-SH cells. Concurrently, the molecular network (MN) derived from the CTF_EA metabolome allows putative identification of flavonol 3-*O*-glycosides, hydrocinnamic acids, and primary metabolites. Molecular docking studies suggest that CTF_EA preferentially targets iNOS, resulting in a decrease in nitric oxide (NO). Furthermore, no toxic effects on normal embryonic development, blood vessel formation, and apoptosis are observed when CTF_EA is tested for in vivo toxicity in zebrafish models. The overall preliminary results suggest the anti-neuroinflammatory and neuroprotective effects of CT and provide scientific support for the efficacy of this medicinal plant at local and traditional levels. However, studies on the targeted isolation of bioactive metabolites, in-depth pharmacological efficacy, and safety in mammalian models are urgently needed to expand our understanding of this plant before it is developed into a promising therapeutic agent for brain-related diseases.

## 1. Introduction

The World Health Organization (WHO) estimates that 792 million people, or 1 in 4 people, in both developed and developing countries, are affected by mental and neurological disorders (MNDs). The global cost for MNDs in 2010 was USD 2.5 trillion and is projected to drastically increase to USD 6.5 trillion by 2030, making mental-related illness the costliest form of chronic disease worldwide [1]. These statistics clearly reveal that there is an urgency to focus on these alarming mental disorders associated with neurological diseases.

Inflammation is an organism’s defense response to biological and chemical stimuli, which, if not properly resolved, can progress into acute or chronic inflammation. Chronic inflammation has been frequently reported to cause diseases such as cancer, asthma, and neurodegenerative diseases [2]. Recently, the concept of inflammation as a common mechanism of disease has been extended to brain-related diseases. Neuroinflammation is characterized by the generation of a set of pro-inflammatory mediators, locally produced by host cells, indicating the engagement of the innate immune system [3]. Recent studies showed that neuroinflammation is an important contributor to the pathogenesis and progression of several brain-related diseases, including Alzheimer’s disease (AD). Several damage signals, such as neurodegeneration, an increase of amyloid-β (Aβ) generation, tau phosphorylation, and cognitive impairment, appear to induce neuroinflammation in AD [4]. AD is the most common cause of dementia, accounting for an estimated 60–70% of cases worldwide. Around 10% of people aged ≥65 years are diagnosed with AD; this figure rises to 32% in those aged >85 years, among whom the annual incidence of AD is estimated at 6.48% [5,6]. Patients with AD present with gradual loss of memory and cognitive functions involving the language, visuospatial, and executive domains. Following the discovery of elevated levels of inflammatory markers in patients with AD, as well as of AD risk genes associated with innate immune functions, inflammation has emerged as a vital player [7,8,9,10,11].

The biological significance of microglia appears to be multifaceted in the central nervous system (CNS); they can exert both neuroprotective and neurotoxic effects. In the normal, healthy brain, microglia can be activated by a wide range of substances, such as lipopolysaccharide (LPS), interferon (IFN)-γ, β-amyloid (Aβ), and α-synuclein. The activation is characterized by a high level of pro-inflammatory factors, including interleukins (IL-1β, IL-6, and IL-18) and tumor necrosis factor (TNF-α), along with an increased production of prostaglandins, nitric oxide (NO), and reactive oxygen species (ROS), which can cause damage to the surrounding neuronal cells [12,13]. NO is a short-lived neurotransmitter and is produced in macrophages, microglia, astrocytes, neurons, and endothelial cells by oxidative deamination of L-arginine to L-citrulline and catalyzed by the NO synthase (NOS) family, with different biological activities depending on where they are synthesized [14,15].

**Figure 1:**
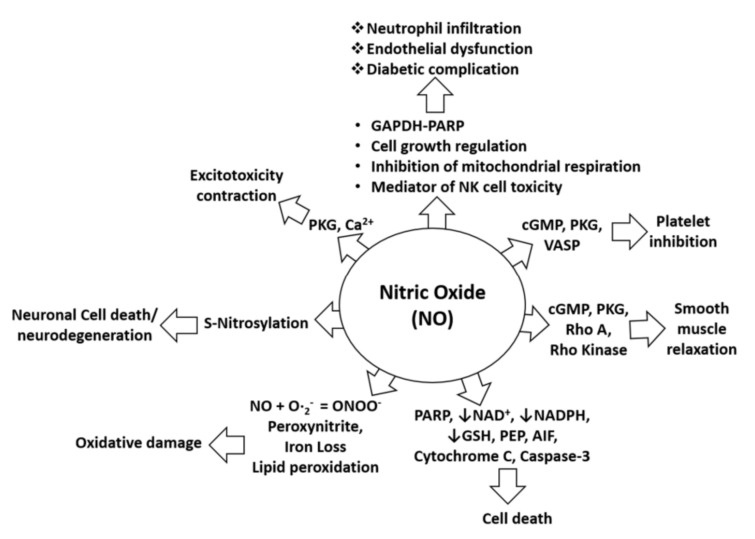
NO in pathophysiological conditions. NO-mediated activation of cGMP, PKG, and VASP can cause platelet inhibition, whereas NO-mediated induction of pro-apoptotic proteins (PARP, AIF, cytochrome C, and cleaved caspase-3) can induce cell death. Furthermore, NO-mediated activation of cGMP, PKG, Rho A, and Rho kinase can alter smooth muscle relaxation, whereas inhibition of NAD, NADPH, and GSH by NO increases cell death. Moreover, lipid peroxidation caused by NO leads to oxidative stress or damage, and S-nitrosylation induced by NO may lead to neurotoxicity or neurodegeneration. In addition, NO-mediated induction of PKG and calcium signaling leads to excitotoxicity and contraction effects. NO is also involved in neutrophil infiltration and endothelial dysfunction through effects on mitochondrial respiration, NK cell toxicity, and activation of the GAPDH-PARP pathway and its functions [26].

Uncontrolled production of NO is identified as one of the most common causes of neurological disorders, including NO-mediated immunomodulation, inflammation, and neurotoxicity. NO is also pathophysiologically associated with oxidative damage, neurodegeneration, diabetic complications, and cell death as it alters the functions and pathways of several target proteins, such as cGMP, cytochrome C, and NADPH, as shown in Figure 1. Specifically, in the pathology of AD, NO plays a critical role in signal transduction pathways important for maintaining brain, vascular, immune, and muscle functions. However, excessive amounts of NO in AD can trigger abnormal posttranslational protein modifications and uncontrolled neurotoxic nitrergic signaling [16,17,18,19]. Cysteine residues of proteins can be directly and reversibly S-nitrosylated by NO [19], leading to protein dysfunction and the promotion of the progression of AD [20,21]. In addition, NO can also react with free radicals, such as superoxide anion, to form peroxynitrite, which accumulatively triggers nitrotyrosination of proteins. This is the irreversible chemical addition of a nitro group to protein tyrosine residues, resulting in the formation of 3-nitrotyrosine (3- NT), which can lead to loss of physiological protein function [22]. Therefore, control of excessive NO formation is a promising, preliminary therapeutic target in many neurologic diseases [23,24,25]. Further, in-depth pharmacological studies are certainly needed to confirm the ability of the drug candidates to treat neurodegenerative diseases, including AD.

The drugs currently used for AD can only temporarily relieve its symptoms; meanwhile, no medication is able to stop or reverse the underlying progress of this disease. The loss of neurons and synapses in the brain is considered the most direct cause of symptoms in AD. Currently, only five drugs have been approved by the Food and Drug Administration (FDA) for clinical treatments of AD. Four drugs, tacrine, rivastigmine, galantamine, and donepezil, are acetylcholinesterase inhibitors (AChEIs), and one, memantine, is an *N*-Methyl-d-aspartate (NMDA) receptor antagonist. However, most drugs cause common adverse effects when consumed, including nausea, diarrhea, vomiting, headache, dizziness, fatigue, weight loss, and muscle weakness [27]. Since then, more research on new medication discovery and the development of a cure for AD with fewer side effects and higher effectiveness continues to be in demand, and traditional plants have become the highlighted alternatives for the treatment of AD, including butterfly pea.

*Clitoria ternatea* Linn. (CT), often known as butterfly pea, is a traditional Ayurvedic plant of the *Fabaceae* family that originated in tropical Asia and has since spread through South and Central America, China, and India, where it has become naturalized. All parts of the plant are used for therapeutic purposes, particularly the roots, seeds, and leaves [28]. The plant extracts are used as an ingredient of Ayurvedic ‘Medhya Rasayana’, which is taken as a brain tonic to improve memory and intelligence. Meanwhile, the flowers of CT are used worldwide as ornamental flowers and are traditionally used as a source of natural food coloring and antioxidants. In addition, the ability of CT to treat CNS disorders, by acting as an anti-stress treatment, antidepressant, and anti-sedative, has been reported [29]. Moreover, the flower extract of CT has shown high potential as an anti-neuroinflammatory agent [30] without acute toxicity and significant hepatoprotective and nephroprotective effects in mice in vivo models [31,32,33,34]. Several studies have been conducted on CT in the past, but little useful information was gathered on the specific metabolites responsible for its medicinal potential. Therefore, to gain a deeper insight into its therapeutic potential, the full spectra of its bioactive metabolites need to be established.

Natural products offer an inexhaustible source for new therapeutic leads. However, the search for new bioactive compounds is hampered by the complexity of the work on the natural product extracts, in which the isolation and characterization of bioactive metabolites are challenging. Furthermore, laborious, time-consuming, and costly bioactivity-guided isolation often yields the known molecules [35]. Recently, a novel bioinformatics approach, namely, molecular networking (MN), was developed to address the complex task of chemical identification of individual metabolites within complex extracts [36,37]. The MN concept is based on the organization and visualization of tandem mass spectrometry (MS^2^) fragmentation data through a spectral similarity map, revealing the presence of homologous MS^2^ fragmentations. As structurally related metabolites share similar fragmentation spectra, their nodes tend to gather and generate a cluster of analogs [35].

In this context, the present study was undertaken to comprehensively phytochemically profile the most active fraction of CT extract, including its neuroprotective and anti-neuroinflammation properties against the LPS-induced microglial-based BV-2 and SK-N-SH neuroblastoma cells, by utilizing an untargeted, tandem-mass-spectrometry-based MN approach. In addition, toxicity profiling using a zebrafish in vivo animal model was also carried out to further confirm the safety of *C. ternatea* flower extract as a promising therapeutic agent for AD and other neuroinflammation diseases.

## 2. Results and Discussion

The methanol extracts of the *C. ternatea* roots (CTR), flowers (CTF), and leaves (CTL) were first tested for their preliminary toxicity to cell viability using the MTT assay, followed by the Griess assay to determine nitric oxide (NO) suppression, in order to assess their neuroprotective, neurotoxicity, and anti-neuroinflammation properties against the LPS-activated BV-2 and SK-N-SH cell lines. It is worth noting that the neuroprotective effects performed demonstrated the ability of the extracts to protect and prevent the death of LPS-induced BV-2 cells [38]. Further, cell-based assays were conducted on the fractions of the most active extract to determine the specific metabolites that are responsible for its medicinal potential.

### 2.1. Biological Evaluation

#### 2.1.1. Effects of CT Extracts on Cell Viability Using Cultured BV-2 Microglial and SK-N-SH Neuroblastoma Cells

**Figure 2:**
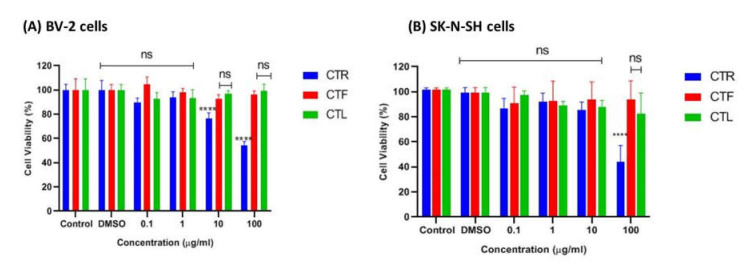
Cytotoxic effect of different parts of *C. ternatea* extracts (CTR, CTF, and CTL) on viability of (**A**) BV-2 microglial and (**B**) human neuroblastoma SK-N- SH cells after 24 h. Percentage cell viability is expressed as mean ± SD of three independent experiments. The value is statistically significant **** (*p* < 0.0001) compared to the untreated cells; ns means not significant.

The tested cells were primarily exposed to the methanolic extracts of CTR, CTF, and CTL for 24 h to ensure no cytotoxic effects on the growth of BV-2 and SK-N-NH cells via MTT assay. According to ISO 10993-5 (2009), a percentage of viable cells greater than 80% is considered non-toxic, a percentage between 80–40% is considered moderately cytotoxic, and a percentage of less than 40% is considered severely cytotoxic. As shown in Figure 2A, the extracts were not cytotoxic to microglial cells at all concentrations tested (0.1 μg/mL, 1 μg/mL, 10 μg/mL, and 100 μg/mL), as the percentage viability of cells was above ~80%, especially for the extracts of CTF and CTL. It is worth noting that the viability of BV-2 cells treated with the extract of CTF was not significantly reduced compared to the control. At the same time, the percentage viability of BV-2 cells gradually decreased (100%, 89%, 84%, 81%) as the concentration of the extract (0.1 μg/mL, 1 μg/mL, 10 μg/mL, and 100 μg/mL) increased for the methanolic extract of CTF in a dose-dependent manner. However, the extract of CTR showed the lowest cell viability (~60%) at the highest concentration (100 μg/mL), indicating a moderate cytotoxic effect on cells.

On the other hand, the extracts of CTL and CTF were not cytotoxic to the SK-N-SH cells at the concentrations tested, with a percent viability of ~80–90%, as shown in Figure 2B. However, the extract of CTR at the highest concentration (100 µg/mL) was moderately toxic to the cells as it had ~50% viable cells compared to the extracts of CTF and CTL (>80%) for both cell lines tested. From this experiment, it can be concluded that extracts of CTL and CTF at concentrations up to 100 μg/mL can be considered safe for both the BV-2 and the SK-N-SH cell lines.

#### 2.1.2. Inhibitory Effects of CT Extracts on NO Secretion in LPS-Induced BV-2 Cells

**Figure 3:**
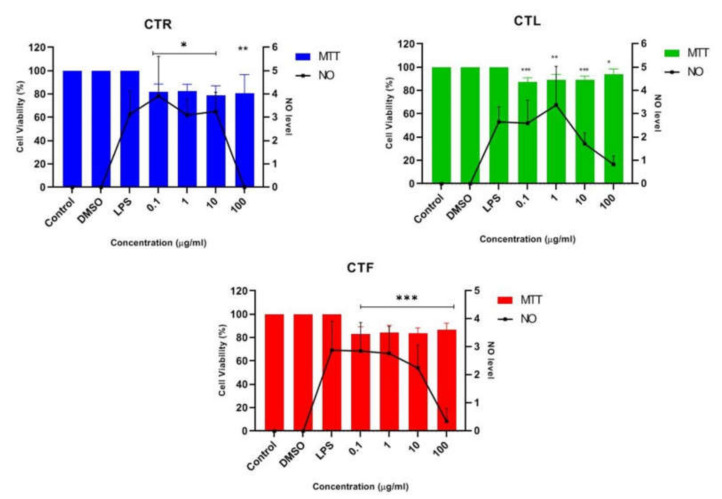
Effect of CTR, CTL, and CTF extracts on NO production in LPS-induced BV-2 cells for 24 h. The NO level is denoted as mean values ± SD, and *n* = 3. The extract showed dose-dependent inhibition of NO release. The value is statistically significant * (*p* < 0.05), ** (*p* < 0.01), and *** (*p* < 0.001) when compared with LPS alone.

The anti-neuroinflammatory effect of the extracts of CT (CTR, CTL, and CTF) on LPS-stimulated inflammation was determined in BV-2 microglial cells at a concentration of 0.1 μg/mL, 1 μg/mL, 10 μg/mL, and 100 μg/mL in triplicate. Cells were pretreated with CT extracts to monitor the NO-inhibitory and neuroprotective effects of LPS-induced BV-2 cells. MTT assays were also performed to ensure that the NO inhibition in the BV-2 cells was not due to cytotoxic activity of CT extracts [39]. Then, the result of pretreatment with the extracts of CT was compared with that of cells induced with LPS alone after 24 h to investigate the significant effect on the suppression of NO, where the NO concentration was calculated using a sodium nitrite (NaNO_2_) standard curve. The efficacy of all extracts on suppressing NO levels and the percentage viability (%) of pretreated BV-2 cells at the tested concentrations are shown in Figure 3. It should be pointed out that the Griess assay could not be performed on the neuroblastoma SK-N-SH cells as a single stimulant, as LPS showed no effect on iNOS levels; however, it could be performed in combination with a stimulant [40].

The results in Figure 3 show that all the concentrations of the extracts studied were able to reduce the synthesis and release of NO. However, a slight decrease in NO concentration was observed for all CT extracts at the concentrations of 0.1 μg/mL, 1 μg/mL, and 10 μg/mL when compared to the untreated cells (LPS alone). The highest concentration of 100 μg/mL was the crucial concentration to inhibit the release of NO and maintain a high percentage of cell viability (~80–90%) for all CTR-, CTL-, and CTF-treated, LPS-induced BV-2 cells. In view of these results, the extract of CTF was selected for further fractionation due to its good neuroprotective and anti-neuroinflammatory properties, as it gradually decreased the NO level in a dose-dependent manner while protecting the LPS-activated, inflamed BV-2 cells. MTT assay disclosed that the number of viable cells was over 80%, confirming that the inhibition of nitric oxide production in LPS-induced BV-2 cells was not due to the cytotoxic effect of CTF extract. Henceforth, it can be tentatively concluded that extracts of CT have a neuroprotective effect on BV-2 while suppressing NO.

#### 2.1.3. Effects of CTF Fractions on Cell Viability Using Cultured BV-2 Microglial and SK-N-SH Human Neuroblastoma Cells

**Figure 4:**
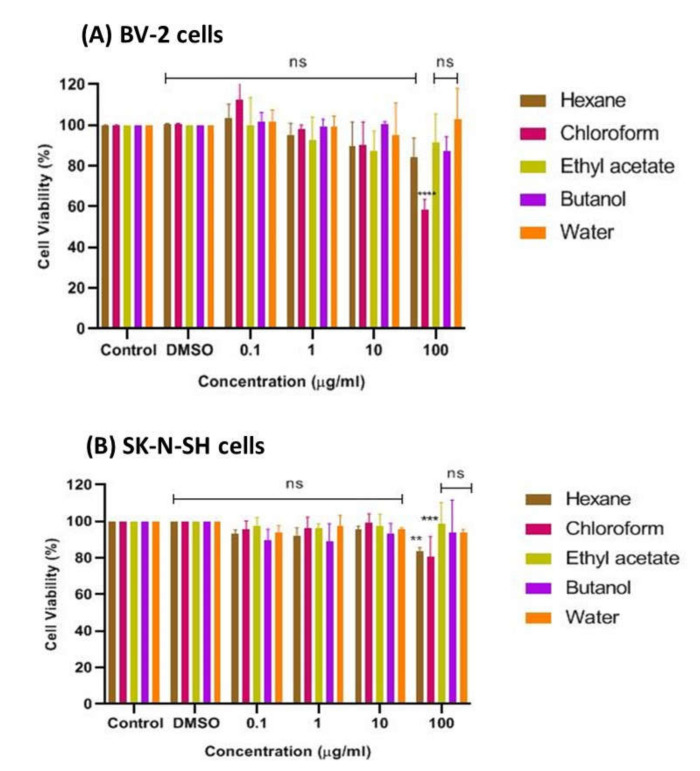
Cytotoxic effect of CTF fractions on viability of (**A**) BV-2 microglial and (**B**) human neuroblastoma SK-N-SH cells after 24 h. Percent cell viability is expressed as mean ± SD of three independent experiments. The value is statistically significant ** (*p* < 0.01), *** (*p* < 0.001), and **** (*p* < 0.0001) compared to the untreated cells; ns means not significant.

The bioactive CTF extract was then successively fractionated into different polarities, including *n*-hexane, chloroform, ethyl acetate, butanol, and water, and tested for cell viability of cultured BV-2 microglia and SK-N-SH human neuroblastoma cells using the MTT assay. The results presented in Figure 4A show that the chloroform fraction had the lowest cell viability, at about 52%, indicating its moderate cytotoxic property and weaker potential to prevent cell death compared to the other fractions of CTF. The findings also show that no fraction exhibited a cytotoxic effect on cells at all concentrations tested, as the percent viability of cells was above ~80%, indicating that all fractions can be considered safe at concentrations up to 100 μg/mL on the BV-2 cells, with the exception of the chloroform fraction. In contrast, the SK-N-SH cell viability, as shown in Figure 4B, indicated that the CTF fractions (ethyl acetate, butanol, and water) were not cytotoxic to cells at all concentrations tested, as the percent viability of cells was above ~80%. However, the hexane and chloroform fractions were moderately cytotoxic toward SK-N-SH cells as they were significantly different when compared with the control. Therefore, it can be assumed that all CTF fractions except the hexane and chloroform fractions were safe for the SK-N-SH neuroblastoma cell line studied at concentrations up to 100 μg/mL.

#### 2.1.4. Inhibition Effects of CTF Fractions on NO Secretion in LPS-Induced BV-2 Cells

The potential anti-neuroinflammatory properties of all fractions of CTF methanolic extract were further investigated by Griess assay on an LPS-induced BV-2 cell line. The results show that the *n*-hexane, chloroform, ethyl acetate, butanol, and water fractions of CTF possessed significant neuroprotective and anti-neuroinflammatory effects in terms of NO suppression of the tested cells.

**Figure 5:**
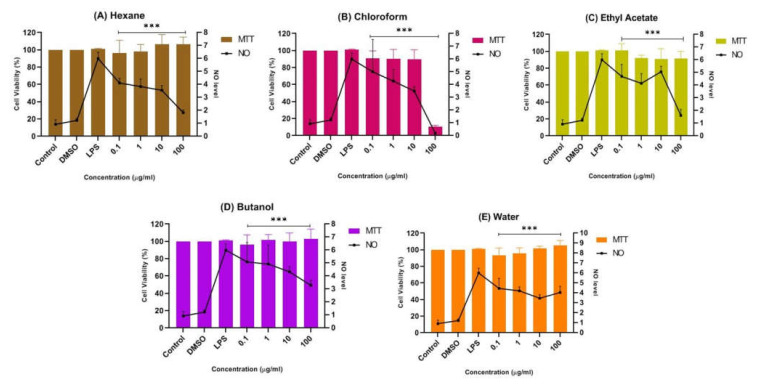
Effect of (**A**) *n*-hexane, (**B**) chloroform, (**C**) ethyl acetate, (**D**) butanol, and (**E**) water fractions of CTF methanolic extract on the production of NO in BV-2 cells after 24 h stimulation by LPS. The NO levels are expressed as mean ± SD of three independent experiments. The extract showed a dose-dependent inhibition of NO release. The values are statistically significant *** (*p* < 0.001) compared to LPS alone.

Figure 5B shows that the chloroform fraction gradually reduced nitrites with increasing concentration, indicating their anti-neuroinflammatory properties. Nevertheless, at the highest concentration of 100 μg/mL, the viability of cells with the chloroform fraction abruptly decreased to below 20%, indicating its neurotoxic properties towards LPS-induced BV-2 microglial cells. It is important to understand that neurotoxic disorders of the nervous system can be due to exposure to chemical or biological agents, resulting in cell death [41]. The neurotoxic effect in the chloroform fraction might have been due to the overactivation of microglial cells. Thus, the suppression of NO in the chloroform fraction at 100 μg/mL can be presumed to be due to the cytotoxic effect of the fraction. Meanwhile, a mild suppression of the NO level was observed in the pretreatment with the butanol and water fractions compared to the untreated cells, as shown in Figure 5D,E. It can be concluded that both fractions also have neuroprotective effects while maintaining a cell viability of above ~90%.

In contrast, Figure 5A,C shows that the pretreatment of BV-2 cells with the hexane and ethyl acetate fractions at all concentrations tested (0.1 μg/mL, 1 μg/mL, 10 μg/mL, and 100 μg/mL) significantly maintained a cell viability of up to ~90%, while reducing the production of NO compared to the non-treated cells. Through the MTT assay results, an increase in viability greater than 100% is unusual, as this represents a possible proliferative or dose-dependent effect compared to the control known as hormesis [42,43,44]. Hormesis is an adaptive dose–response relationship characterized by stimulation at low dose and inhibition or toxic effect at high dose [45]. Thus, the hexane fraction could have exhibited an adaptive dose–response relationship as cell viability was above 100% at 10 µg/mL and 100 µg/mL compared to the control. Although the viable cells were not toxic in Griess assay and were significantly suppressing the NO level, the hexane fraction in MTT assay was moderately cytotoxic towards SK-N-SH cells (Figure 4B).

Therefore, the ethyl acetate fraction of CTF (denoted as CTF_EA) was selected for comprehensive profiling and identification of bioactive phytochemicals responsible for its anti-neuroinflammatory and neuroprotective properties, as it did not cause any cytotoxic effects toward BV-2 cells and SK-N-SH cells in MTT assay. It also had a significant effect in suppressing the NO level on BV-2 cells. This task was performed by using advanced LC–MS/MS in combination with cheminformatics and a molecular network (MN).

### 2.2. Metabolic Profiling of Ethyl Acetate Fraction of CTF (CTF_EA) by the Untargeted, Tandem-Mass-Spectrometry-Based (UHPLC–MS/MS) Molecular Networking

**Figure 6:**
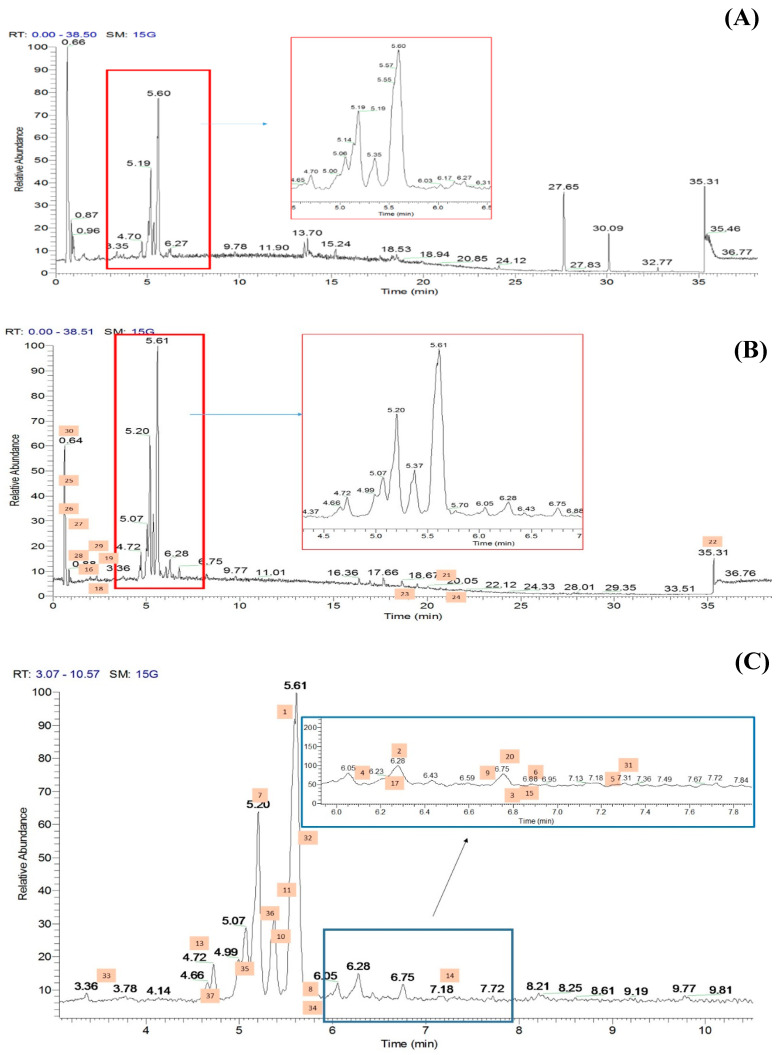
Total ion chromatograms (TICs) of the ethyl acetate fraction of flower methanolic extract of *C. ternatea* (CTF_EA) in (**A**) positive mode, (**B**) negative mode, and (**C**) negative modes in between 3rd to 10th minutes. The number above each peak represents peak numbers, corresponding to the peak numbers in Table 1.

The methanolic flowers of CT (CTF) were extracted using an accelerated solvent extraction technique and were further fractionated into different polarity solvents including ethyl acetate to yield the most bioactive fraction, CTF_EA. The CTF_EA was analyzed by UHPLC–MS/MS, and the obtained fragmentation data from tandem LC–MS/MS were used to generate a molecular network (MN) in order to establish the detailed phytochemical composition of the flower metabolome, particularly of the ethyl acetate fraction. More than 300 MS/MS spectra over the mass range of *m/z* 120 to 1500 were generated in both positive and negative ion modes, where most of the metabolites were eluted between the fourth and seventh minutes. The respective total ion chromatograms (TICs) obtained are presented in Figure 6.

The chemical space of CTF_EA was further analyzed in detail by mapping its structurally related compounds into the MN. To achieve this, the acquired, large datasets of MS/MS fragmentation spectra were organized as MNs by using the classical workflow available on the GNPS experimental workflow (http://gnps.ucsd.edu) accessed on 16 June 2021. The MNs generated from the positive and negative modes’ mass spectral data of the CTF_EA are attached in Supplementary Materials (see Figure S1). Each representative MS/MS spectrum of the detected molecular ions was visualized as a node (red circle), while the edge (gray line) connecting the nodes indicated that they possessed a homologous MS/MS fragmentation pattern, hence, allowing them to be mapped together within the same cluster (node ≥ 2). All the identified metabolites with respect to their retention time (RT) and MS/MS data are as listed in Table 1.

**Figure 7:**
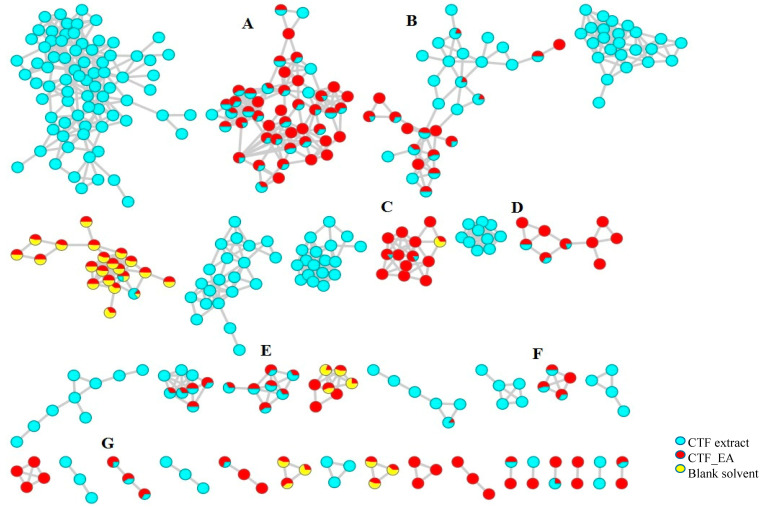
Full visualization of the molecular network in negative ion mode of the flower extract (CTF) and ethyl acetate fraction (CTF_EA) of *C. ternatea*. The annotated classes of metabolites are: (**A**) flavonol 3-*O*-glycosides, (**B**) hydrocinnamic acids and derivatives, (**C**) glycerophospholipid, (**D**) amino acids, (**E**) carbohydrates, (**F**) mono-methoxylflavonol 3-*O*-glycoside and (**G**) saccharolipid.

**Figure 8:**
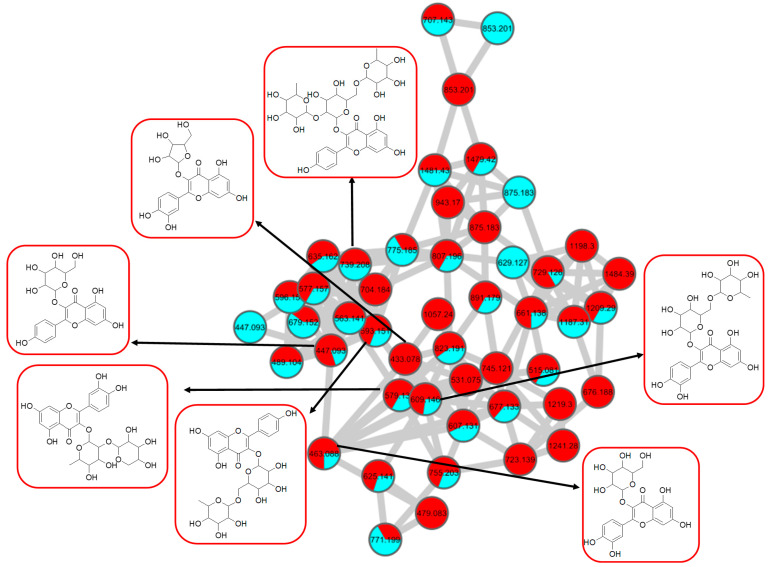
Flavonol 3-*O-*glycoside (cluster A in negative mode) from the full molecular network (MN) of ethyl acetate fraction of flower *C. ternatea* (CTF_EA) extract. The metabolites in red boxes were annotated based on the GNPS library matching.

The LC–MS/MS analyses were performed in both positive and negative modes, and most of the metabolites responsible for the targeted bioactivities were better ionized in the negative mode, as depicted in Figure 7. Therefore, the identification of compounds was based on their full MS and MS/MS spectra obtained in the negative ion mode. To get a better overview of the distribution and identification of the bioactive metabolites, we compiled the CTF_EA mass spectra in a CTF extracts MN (see Figure 8). The following subsections discuss the annotation of the metabolites of selected clusters A, B, and F, as mapped in the network (other clusters C, D, E, and G are appended along with their respective fragmentation pathways in the Figures S2–S9 in Supplementary Materials).

#### 2.2.1. Flavonol-3-*O*-glycoside

The analysis of negative ion mode MS/MS spectral data for cluster A (Figure 8) showed the presence of mono-, di-, tri-, and acylated flavonol *O*-glycoside, specifically kaempferol- and quercetin-type *O*-glycoside. The glycosylated position was confirmed at 3-OH position through the aglycone homolytic cleavage, [Y_o_-H]^●^^−^ and heterolytic cleavage, [Y_o_]^−^ by comparing the relative abundance of both fragmentation patterns between the generated MS/MS spectral data with known flavonol-3-*O*-glycoside in the literature [46]. Meanwhile, several flavonol-3-*O*-glycosides present in CTF_EA were putatively annotated by GNPS spectral library matching. The nodes with a precursor ion at *m/z* 447.1345 [M-H]^−^, *m/z* 593.1503 [M-H]^−^, and *m/z* 739.1042 [M-H]^−^ were annotated as kaempferol-3-*O*-glucoside (**2**), kaempferol-3-*O*-rutinoside (**1**), and kaempferol-3-*O*-(2-rhamnosyl) rutinoside (**7**), respectively. The nodes with a precursor ion at corresponding *m/z* 433.078 [M-H]^−^, *m/z* 579.1364 [M-H]^−^, *m/z* 609.1461 [M-H]^−^, and *m/z* 463.0887 [M-H]^−^ were identified as avicularin (**8**, scientifically identified as quercetin 3-α-*L*-arabinofuranoside), quercetin-3-*O*-deoxyhexosyl (1–2) pentoside (**9**), quercetin-3-*O*-rutinoside (**10**, commonly known as rutin), and isoquercetin (**11**).

Further analyses of MS/MS fragmentation patterns in other databases, the Metabolomics Workbench and PubChem, permitted the annotation of six known compounds, including kaempferol-3-*O*-α-*L*-rhamnosyl-(1->2)-*O*-*L*-rhamnoside (**5**), kaempferol 3-(6″-acetylglucoside) (**3**), kaempferol 3-(6G-malonylneohesperidoside) (**4**), kaempferol 3-*O*-(4″-*O*-acetyl) rutinoside (**6**), quercetin 3-(2G-glucosylrutinoside) (**12**), and manghaslin (**13**). The precursor ions were found at the respective nodes of *m/z* 577.269 [M-H]^−^, *m/z* 489.1045 [M-H]^−^, *m/z* 679.1527 [M-H]^−^, *m/z* 635.162 [M-H]^−^, *m/z* 771.1786 [M-H]^−^, and *m/z* 755.2035 [M-H]^−^.

**Figure 9:**
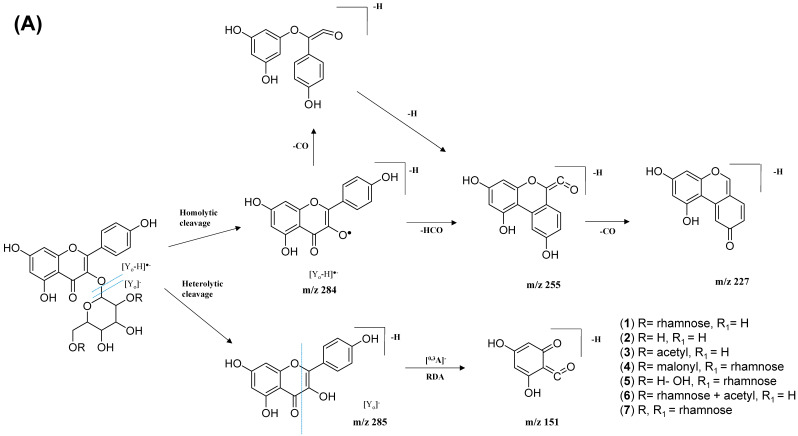

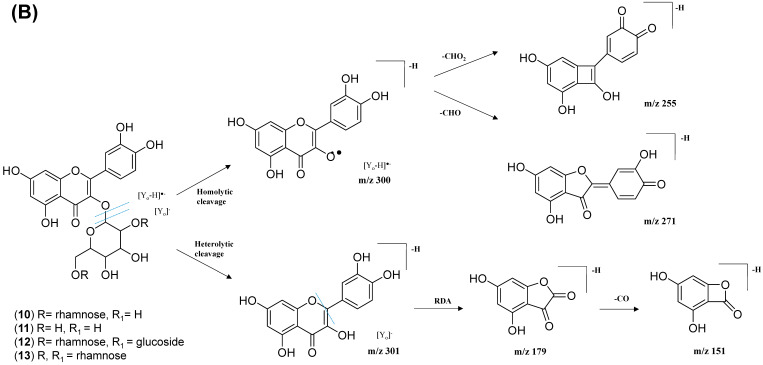
Proposed fragmentation mechanism of (**A**) kaempferol 3-*O*-glycoside and (**B**) quercetin 3-*O*-glycoside in negative ion mode.

The kaempferol and quercetin derivatives were distinguished through the main aglycone products that were generated via the homolytic and heterolytic cleavage of sugar moiety, as shown in the proposed main fragmentation pathway. Figure 9A illustrates the formation of aglycone ions at *m/z* 284 [M-H]^−^ (base peak) and *m/z* 285 [M-H]^−^, which was then further fragmentated to yield ions at *m/z* 255 [M-H]^−^, *m/z* 227 [M-H]^−^, and *m/z* 151 [M-H]^−^ and, thus, positively confirmed the annotated metabolites as a kaempferol. Contrarily, the main aglycone product ions observed at *m/z* 301 [M-H]^−^ and *m/z* 300 [M-H]^−^ (base peak), followed by the fragments at *m/z* 271 [M-H]^−^, *m/z* 255 [M-H]^−^, and *m/z* 151 [M-H]^−^, were exclusive attributed to the quercetin scaffold. The proposed fragmentation mechanism for quercetin 3-*O*-glucoside is visualized in Figure 9B.

#### 2.2.2. Hydrocinnamic Acids and Derivatives

**Figure 10:**
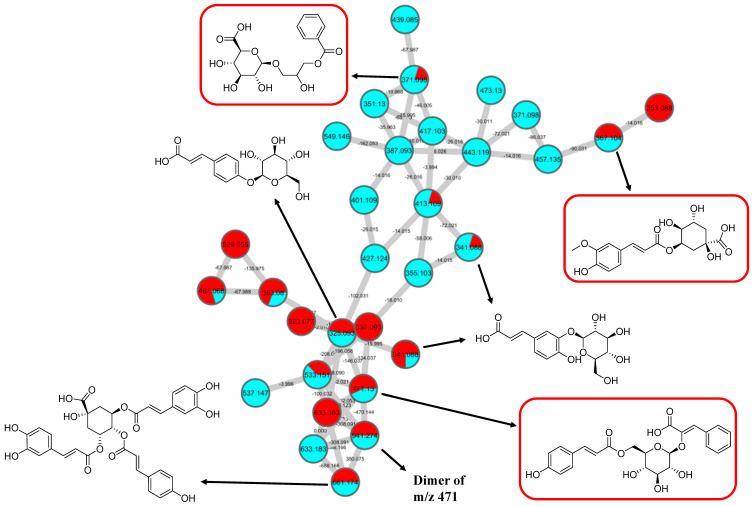
Hydrocinnamic acids and derivatives (cluster B in negative mode) from the full molecular network (MN) of ethyl acetate fraction of flower *C. ternatea* (CTF_EA) extract. The metabolites in red boxes were annotated based on the GNPS library matching.

GNPS spectral library matching to spectral nodes in cluster B with precursor ions at *m/z* 371.096 [M-H]^−^, *m/z* 471.13 [M-H]^−^, and *m/z* 367.1036 [M-H]^−^ enabled the annotation of three metabolites, namely, 3-(benzoyloxy)-2-hydroxypropyl β-d-glucopyranosiduronic acid (**16**), 3-phenyl-2-[(2*S*,3*R*,4*S*,5*S*,6*R*)-3,4,5-trihydroxy-6-[[(*E*)-3-(4-hydroxyphenyl)prop-2-enoyl]oxy-methyl]oxan-2-yl]oxyprop-2-enoic acid (**14**), and feruloylquinic acid isomer (**17**), as illustrated in Figure 10. The MS/MS fragmentation patterns of these structural compounds were studied and found to be compatible with the hydrocinnamic acids and derivatives. The fragmentation ions of coumaroyl, caffeoyl, and feruloyl were observed in cluster B at *m/z* 163, *m/z* 179, and *m/z* 193, respectively.

**Figure 11:**
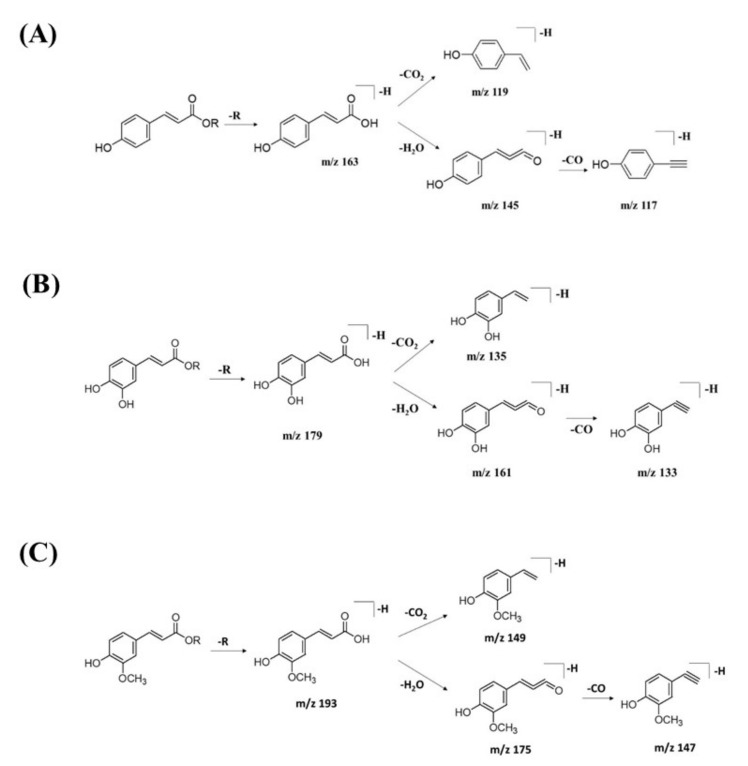
Fragmentation pathway for (**A**) coumaroyl, (**B**) caffeoyl, and (**C**) feruloyl derivatives.

The MS/MS spectral data of 3-(benzoyloxy)-2-hydroxypropyl β-D-glucopyranosiduronic acid (**16**) at *m/z* 371.096 [M-H]^−^ revealed that the benzoyl fragment ion at *m*/*z* 163 was caused by the internal cleavage of the glucoronic acid moiety. The successive loss of 46 Da (CO + H_2_O) was consistent with the presence of a unit of the carboxyl group within the parent structure, along with the characteristic fragment ion at *m/z* 119 (due to internal CO_2_ cleavage from the benzoyl intermediate *m/z* 163). The fragmentation mechanism for the node containing the precursor ion at *m*/*z* 471.13 [M-H]^−^ was found to be 3-phenyl-2-[(2S,3R,4S,5S,6R)-3,4,5-trihydroxy-6-[[(E)-3-(4-hydroxyphenyl)prop-2-enoyl]oxymethyl]oxan-2-yl]oxyprop-2-enoic acid (**14**), which matched the characteristic fragment ions of coumaroyl at m/z 163 for [(M-H-glucosyl unit (162Da)-benzoic acid (147Da)]^−^ along with two major fragment ions at *m*/*z* 145 and *m*/*z* 119 (Figure 11A). Moreover, a node with the precursor ion at *m/z* 941.2737 [M_2_-H]^−^ was putatively characterized as a dimer of 3-phenyl-2-[(2*S*,3*R*,4*S*,5*S*,6*R*)-3,4,5-trihydroxy-6-[[(*E*)-3-(4-hydroxyphenyl)prop-2-enoyl]-oxy-methyl]oxan-2-yl]oxyprop-2-enoic acid (**15**). In particular, it showed a characteristic fragment ion of [M_1_] at *m/z* 471 and the dehydrated coumaroyl at *m/z* 163, followed by dehydration and decarboxylation at *m/z* 145 and *m/z* 119, respectively. Based on the Metabolomic Workbench database, the deprotonated ion at *m/z* 661.1782 [M-H]^−^ was identified as 3,5-di-*O*-caffeoyl-4-*O*-coumaroylquinic acid (**20**). The major coumaroyl fragment ion yielded at *m/z* 163, resulting from the loss of the quinic acid moiety (175 Da), was followed by the successive fragment ions *m/z* 145 and *m/z* 119.

Detailed examination of the MS/MS spectral data of the other nodes revealed the identification of known phenolic acid glycosides at *m/z* 325.1843 [M-H]^−^ for *p*-coumaric acid 4-*O*-glucoside (**19**) based on the characteristic fragment ions of coumaric acid at *m/z* 163 [(M-H)-sugar unit (162 Da)] ^−^, *m/z* 145, and *m/z* 119. Meanwhile, *m/z* 341.0877 [M-H]^−^ was annotated as caffeic acid-*O*-glucoside (**18**), with a major peak observed at *m/z* 179, representing caffeic acid, followed by decarboxylation at *m/z* 135 (Figure 11B). Another GNPS-matched node with precursor ion at *m/z* 367.1036 [M-H]^−^ was characterized as feruloylquinic acid isomer (**17**) based on the fragment ion for a dehydrated quinic acid moiety at *m/z* 175 [M − feruloyl-H_2_O]^−^, resulting from the loss of the feruloyl moiety (193 Da) and the subsequent decarboxylation, giving the fragment ion at *m/z* 149 (Figure 11C).

#### 2.2.3. Mono-Methoxylflavonol 3-*O*-glycoside

**Figure 12:**
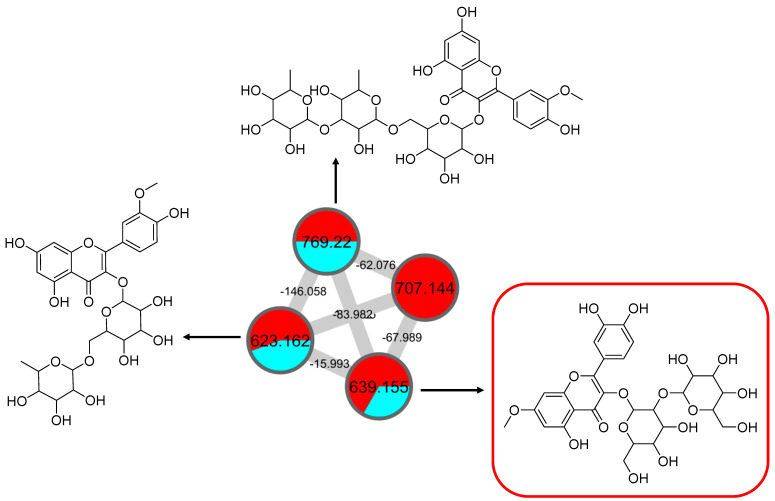
Mono-methoxyl flavonol 3-*O*-glycosides (cluster F in negative mode) from the full molecular network (MN) of ethyl acetate fraction of flower *C. ternatea* (CTF_EA) extract. The metabolites in the red box were annotated based on GNPS library matching.

**Figure 13:**
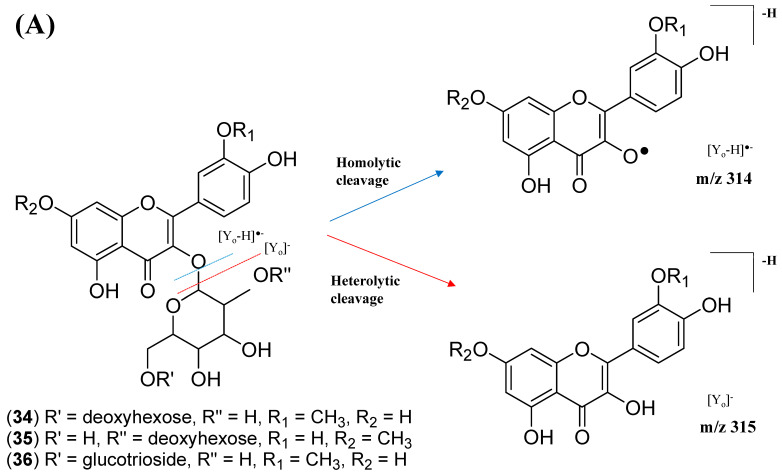

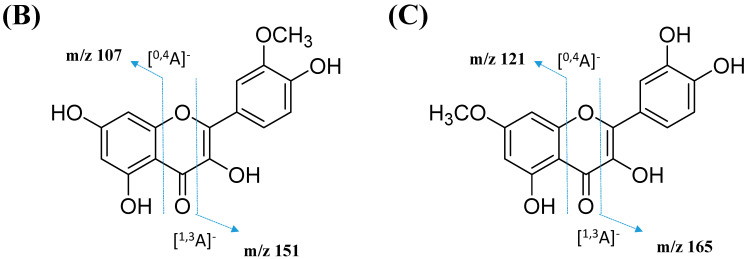
Proposed fragmentation mechanism in the MS/MS spectra recorded for the (**A**) mono-methoxyl group in negative ion mode of (**B**) isorhamnetin and (**C**) rhamnetin.

This cluster F indicates the presence of mono-methoxylflavonol 3-*O*-glycoside and is shown in Figure 12. Analysis of MS/MS spectral data of all dereplicated metabolites confirmed the loss of glycosyl moiety of 308 Da with two predominant fragments at *m/z* 315 and *m/z* 314. Based on the proposed glycosidic fragmentation of flavonol 3-*O*-glycoside in Figure 13A, the position of the glucoside with the higher intensity of *m/z* 314 (3-OH glycoside) was confirmed when compared to the low intensity of *m/z* 315 (7-OH glycoside).

The node with the precursor ion at *m/z* 623.1410 [M-H]^−^ was identified as isorhamnetin-3-galactoside-6″-rhamnoside (**34**), while *m/z* 639.2764 [M-H]^−^ was annotated as rhamnetin-3-*O*–gentiobioside (**35**), which was consistent with the GNPS library. Although there is no general approach for analyzing the exact position of the methoxy substituent on the flavonol core structure, the masses of the A-ring fragments can be used to determine whether the methoxy group is attached to the A or B ring [47]. The existence of isorhamnetin and rhamnetin as the aglycone units of these compounds can be further confirmed based on the formation of fragment ions that arise from the cleavage of ring C of the mono-methoxylflavonol at positions 1 and 3, via the retro-Diels–Alder (RDA) mechanism, [^1,3^A]^−^ and the fragment ion of [^0,4^A]^−^. Specifically, these fragment ions appeared at *m/z* 107 [^0,4^A]^−^ and 151 [^1,3^A]^−^ for isorhamnetin, while at *m/z* 121 [^0,4^A]^−^ and 165 [^1,3^A]^−^ for rhamnetin. A further loss of a 15 Da (CH_3_), accompanied by CO (28 Da) and HCO (29 Da), indicated the presence of methoxy group in their parent structures [30,31,32]. The spectra show cross-ring cleavage of the mono-methoxyl sub-structure, which can be seen from the proposed fragmentation mechanism in Figure 13B,C. In addition, a node with a precursor ion at *m/z* 769.2203 [M-H]^−^ was putatively characterized as a tri-glucoside isorhamnetin, namely 3-((6-(((3,5-dihydroxy-6-methyl-4-((3,4,5-trihydroxy-6-methyltetrahydro-2*H*-pyran-2-yl)oxy)-tetra-hydro-2*H*-pyran-2-yl)oxy)-methyl)-3,4,5-trihydroxytetrahydro-2*H*-pyran-2-yl)oxy)-5,7-di-hydroxy-2-(4-hydroxy-3-methoxy-phenyl)-4*H*-chromen-4-one (**36**). The fragment ion showed the loss of a sugar unit, resulting in the mass fragment ion of *m/z* 605. The fragment ion at *m/z* 314 as the base peak confirmed the resemblance of the structure to isorhamnetin after the loss of another sugar moiety.

### 2.3. Molecular Docking

Nuclear factor kappa-β (NF-κβ) and mitogen-activated protein kinase (MAPK) are known to be crucial transcription factors regulating the expression of iNOS and COX-2, respectively. Disruption of these signaling pathways may downregulate the expression of iNOS and COX-2, thereby, reducing the production of NO. To determine the possible mechanism of action of CTF_EA, molecular docking analyses were firstly performed to investigate the binding energies between three major, annotated flavonol 3-*O*-glycosides, compounds 1, 7, and 10, and several target enzymes, including p38 (PDB ID: 2GTN), ERK-2 (PDB ID: 3C9W), JNK (PDB ID: 3O2M), COX-2 (6COX), and iNOS (3E6T).

The binding energies were used as a measure to compare the binding affinity of compounds 1, 7, and 10 with their corresponding co-crystallized ligand of different enzymes, as shown in Table 2. Among the target enzymes, compounds 1, 7, and 10 showed favorable binding energies of −10.11 kcal/mol, −8.78 kcal/mol, and −8.33 kcal/mol, respectively, which were very similar to the binding energy of the co-crystallized ligand of iNOS, −7.64 kcal/mol. Therefore, the discussion will focus on the molecular docking of the main active metabolites 1, 7, and 10 in the iNOS binding pocket.

**Figure 14:**
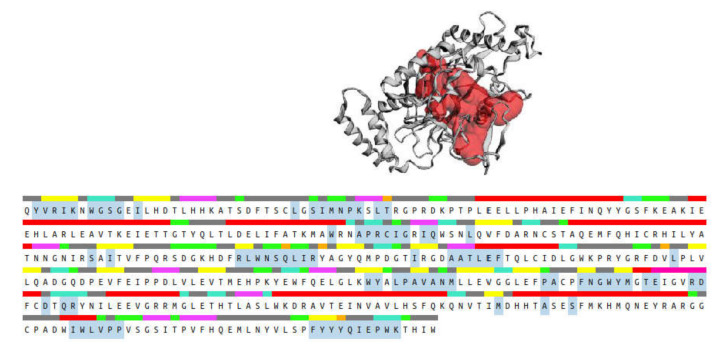
Binding pocket (red color) from the CASTp 3.0 tool, along with the sequence showing the residues highlighted in blue that form the binding pocket.

The CASTp 3.0 provides binding pockets containing amino acid residues that may be responsible for protein–ligand interactions, and it predicted only one binding pocket of iNOS enzyme (PDB ID: 3E6T) with an area of 1893.614 Å^2^ and volume of 1887.502 Å^3^ (Figure 14). Key residues (Ser112, Arg193, Cys194, Trp188, Ser236, Gln257, Arg260, Tyr341, Pro344, Val346, Phe363, Asn364, Gly365, Trp366, Tyr367, Glu371, Asp376, Arg382, Arg375, Trp455, Ile456, Trp457, Phe470, His471, and Tyr485) were determined by crossing the results from CASTp 3.0 server and DSV 19.1. These key residues interact mainly with small molecules and might contribute to the inhibition of NO production.

To validate the accuracy and reliability of the AutoDock 4.2.6 docking protocol for the present purpose, the co-crystallized 1A2905 was re-docked in the binding pocket of the iNOS enzyme obtained from the CASTp 3.0 server, and the re-docked position was compared with the position of the crystal structure by calculating the root mean square deviation (RMSD). This is a typical method to compare the structural similarity between two superimposed structures [48] (see Figure S10 in Supplementary Materials), where the successful scoring function is the one with the RMSD value of ≤2.0 Å [49]. In this study, the RMSD value of the re-docked 1A2905 from the crystal structure was 0.4662 Å, indicating our docking methods were valid for the given structures and AutoDock 4.2.6.

Molecular docking analysis was performed to investigate the intermolecular interactions between major, annotated flavonol 3-*O*-glycosides, compounds **1**, **7**, and **10**, and the iNOS enzyme. The results showed that all residues interacting with compounds **1**, **7**, and **10** (Table 3) were included in the blue, highlighted sequence (Figure 14), predicting that compounds **1**, **7**, and **10** might directly interact with the cavity residues of the iNOS enzyme.

**Figure 15:**
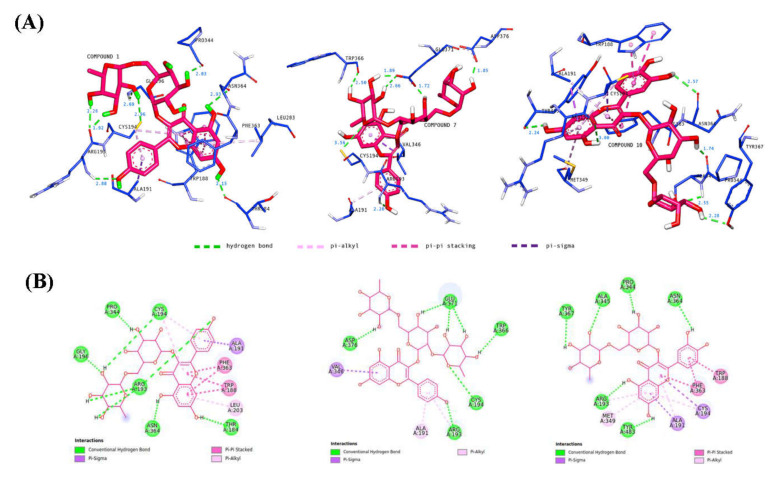
Binding interactions of major active metabolites **1**, **7**, and **10** against the iNOS enzyme with (**A**) 3D and (**B**) 2D representations. Each hydrogen distance (Å) is labeled in blue color near its respective hydrogen bond. For clarity, only ligands and interacting residues are shown.

It is well understood that binding affinity is majorly affected by non-covalent intermolecular interactions, such as hydrogen bonding, electrostatic interactions, and hydrophobic and Van der Waals forces, between the two molecules. In this present study, the hydroxyl groups of rutinose moiety led to the formation of 13 hydrogen bonds between compounds **1**, **7**, and **10** and Arg193, Cys194, Gly196, Pro344, Trp366, Ala345, Tyr367, Glu371, and Asp376 of iNOS residues. Only one hydrogen bond was formed directly between the oxygen of rutinose (compound **7**) and the Cys196 residue (Figure 15). The hydroxyl functionality of the flavonoids (kaempferol and quercetin) contributed to the formation of seven hydrogen bonds, with three hydrogen bonds formed between the hydroxyl groups of the phenyl ring containing residues Arg193 and Asn364, and four hydrogen bonds formed between the hydroxyl groups of the 4*H*-1-benzopyran-4-one containing residues Thr184, Arg193, Asn364, and Tyr483. Compounds **1**, **7**, and **10** also interacted with the active site of iNOS by forming hydrophobic interactions, including π–alkyl (Ala191, Arg193, Cys194, Met149, Leu203), π–sigma (Ala191, Cys194, Val346), and π–π stacked (Trp188, Phe363). Most of these hydrophobic interactions were formed between the iNOS residues and the flavonoids scaffold of compounds **1**, **7**, and **10** due to the presence of a π electron cloud over two aromatic groups (phenyl and dihydroxybenzene attached to the γ-pyrone ring) and C=C moiety in the γ-pyrone of the flavonoids. Overall, compounds **1**, **7**, and **10** exhibited lower binding energy than the co-crystallized ligand. Therefore, it is suggested that compounds **1**, **7**, and **10** may preferentially target iNOS and, thereby, reduce the production of NO.

### 2.4. In Vivo Toxicity Test in Zebrafish Embryos

It should be noted that natural-based medications or supplements are not exempt from adverse health effects, even though natural products are often touted as safe and effective in a wide range of doses [50]. Therefore, toxicological evaluation of the most active fraction, CTF_EA, is an essential step to determine the safe levels for its practical usage in healthcare. In recent years, zebrafish has been extensively used for toxicity profiling due to its advantageous characteristics such as small size, rapid external development, high reproductive rate, and high physiological similarity (organ systems and tissues) to humans [51]. In addition, most of the functional domains of human proteins and zebrafish are highly conserved, which means that the small molecules discovered by using the zebrafish model should have a similar or closely related target in humans [52]. Moreover, testing on a zebrafish model can also be completed in a short timeframe, which is extremely valuable, and the embryos exhibit good dose response to toxicity [53].

**Figure 16:**
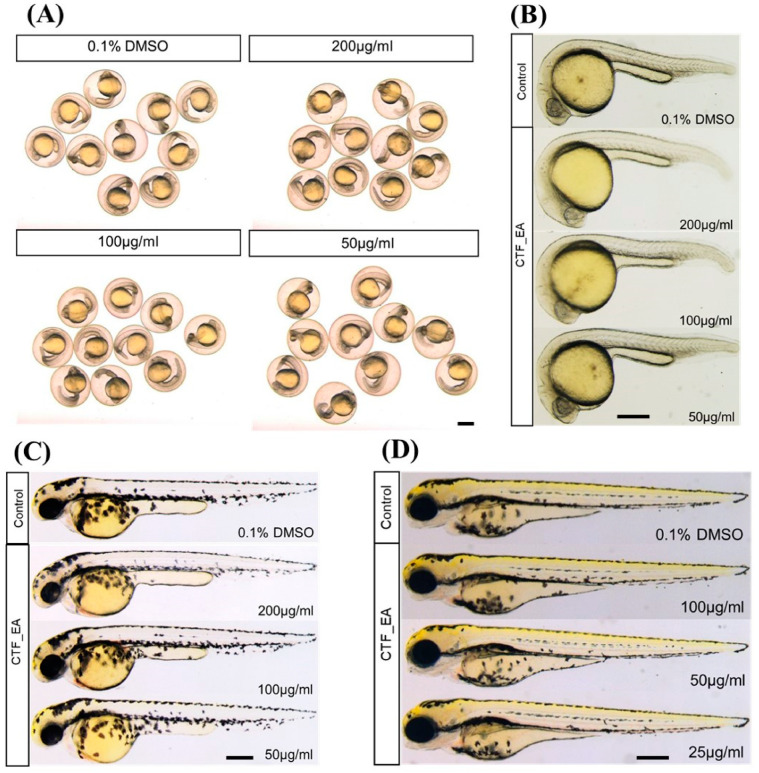
Morphological characteristics of zebrafish larvae at different developmental stages of (**A**) 24 hpf, (**B**) 24 hpf (dechorionated zebrafish), (**C**) 48 hpf, and (**D**) 72 hpf with CTF_EA fraction at concentrations of 200 µg/mL, 100 µg/mL, and 50 µg/mL. The changes in body size, yolk expansion, somite boundary, and pigment cell development, as well as heart rate and blood circulation (data not shown), were observed and measured. Scale bar, 200 µm.

To investigate the developmental defects in zebrafish caused by repeated exposure to CTF_EA, we treated zebrafish embryos with different concentrations (200 µg/mL, 100 µg/mL, and 50 µg/mL) from the early developmental stage of 4 hpf and observed the development of zebrafish successively at 24 hpf, 48 hpf, and 72 hpf. The results showed that most embryos survived at a high concentration of 200 µg/mL up to 72 hpf without significant signs of toxicity and abnormalities after treatment with CTF_EA. There were no detectable changes in body size, yolk expansion, somite boundary, heart rate, blood circulation, and pigment cell development of treated larvae compared to control (Figure 16).

**Figure 17:**
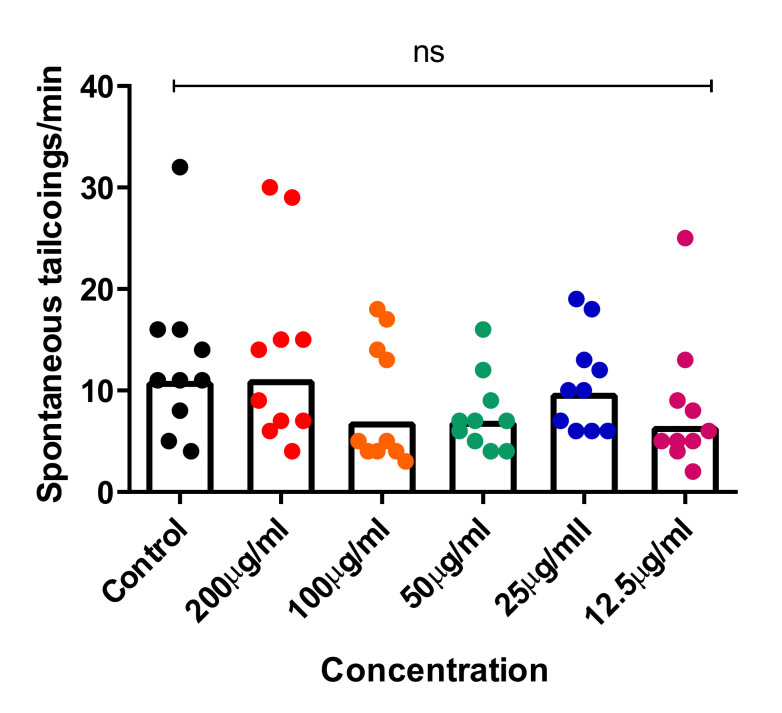
Tail-coiling rate in zebrafish embryos exposed to CTF_EA at different concentrations (200 µg/mL, 100 µg/mL, 50 µg/mL, 25 µg/mL, and 12.5 µg/mL) at 24 hpf developmental stage. Each dot represents the number of spontaneous tail coils per individual animal (*n* = 10 for each concentration). Mean values were expressed as bar graphs; ns means not significant.

Recent studies showed that the spontaneous tail-coiling movement of zebrafish embryos is a powerful tool to assess the integrative effects of exposed chemicals/toxicants at the whole-organism level, including during nervous system development. This simple pattern of movement in zebrafish derives from a basic neural circuit located in the spinal cord nervous system and can be altered by a wide range of structurally diverse neurotoxic compounds, resulting in hypo- or hyperactivity in exposed zebrafish embryos [54,55,56]. To further assess the neurotoxic effects of CTF_EA, we simultaneously analyzed early tail-coiling activity in the tested zebrafish embryos. Ten embryos per concentration (200 µg/mL, 100 µg/mL, 50 µg/mL, 25 µg/mL, and 12.5 µg/mL) were placed in a 24-well plate at the developmental stage of 22 hpf and incubated for 2 h. The average number of tail-coiling movements in one minute was counted at 24 hpf for each concentration along with a control of 0.1% DMSO. As can be seen in Figure 17, there was no significant change in the mean spontaneous tail-coiling activity of the embryos treated with CTF_EA compared to that of the control.

**Figure 18:**
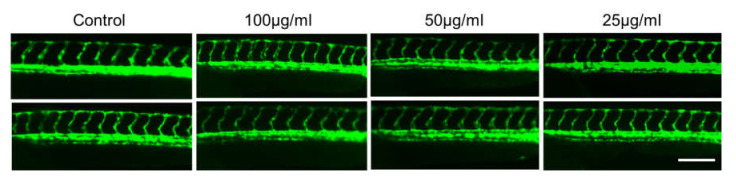
Effects of CTF_EA at different concentrations of 100 µg/mL, 50 µg/mL, and 25 µg/mL on blood vessel development in transgenic zebrafish, *Tg(kdrl:egfp)* at 30 hpf. Vasculogenesis and angiogenesis in the trunk region were normal in both control and CTF_EA-treated zebrafish. Scale bar, 200 µm.

It has been established that the development of vascular anatomy in zebrafish bears a strong resemblance to other vertebrates because of its optical transparency and the early growth of the circulatory system [57]. Many studies in recent years showed that the molecular and morphological mechanisms involved in vascular development are evolutionarily well conserved between zebrafish and mammals. To investigate the toxic effects of CTF_EA on blood vessel development in zebrafish larvae, we used a *Tg(kdrl:egfp)* transgenic zebrafish line that expressed green fluorescent protein (GFP) in its vascular endothelial cells. Zebrafish larvae were incubated at the 10 hpf stage for a day with CTF_EA at various concentrations of 100 µg/mL, 50 µg/mL, and 25 µg/mL and evaluated at 30 hpf under a fluorescence microscope. As shown in Figure 18, even at a concentration of 100 µg/mL CTF_EA, the active phytochemicals showed no adverse effects on vasculogenic and angiogenic phenotypes.

**Figure 19:**
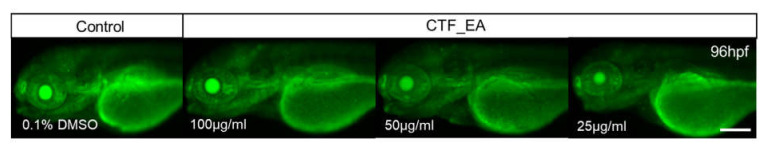
Visualization of apoptosis on CTF_EA-treated zebrafish larvae at different concentrations of 100 μg/mL, 50 μg/mL, and 25 μg/mL, respectively, using the vital fluorescent acridine orange staining. Signals in yolk and lens are autofluorescence. Scale bar, 200 µm.

In addition, we wanted to verify whether the bioactive CTF_EA could induce apoptosis or cell death in zebrafish larvae. Analysis of cell death in zebrafish larvae after treatment with bioactive CTF_EA fraction is important for evaluating the relative toxicity. Morphologically, cell death is classified into distinct patterns in the dying cells, such as cell shrinkage, blebbing in the plasma membrane, nuclear condensation, and fragmentation [58]. Using the vital fluorescent dye acridine orange, an efficient method for detecting apoptotic cells in assayed animals, we did not observe any negative effect on apoptosis after treating the zebrafish larvae with different concentrations of CTF_EA (100 µg/mL, 50 µg/mL, and 25 µg/mL) compared to the control, as shown in Figure 19.

## 3. Material and Methods

### 3.1. Reagents and Materials

All chemicals, solvents, and reagents used for extraction, fractionation, in vitro biological, and in vivo toxicity evaluations were purchased from Sigma Aldrich (St. Louis, MO, USA), Merck (Branchburg, NJ, USA), Systerm (Shah Alam, Malaysia), Nacalai Tesque (Kyoto, Japan), Molecular Probes (Eugene, OR. USA), Invitrogen (Waltham, MA, USA), Fisher Scientific (Waltham, MA, USA), San Francisco Bay Brand (Newark, NJ, USA) and Sera (Grapevine, TX, USA) and used without further purification. The BV-2 mouse-based microglial and SK-N-SH human neuroblastoma cells of American Type Culture Collection (ATCC, Rockville, MD, USA) were provided by Prof. Dr. Johnson Stanslas of the Pharmacotherapeutics Lab, Faculty of Health and Sciences, Universiti Putra Malaysia.

### 3.2. Plant Material

The plant name *Clitoria ternatea* Linn. (CT) was verified by http://www.theplantlist.org (accessed date: 28 January 2022). CT was planted and obtained from Taman Pertanian Universiti (TPU), Universiti Putra Malaysia, Serdang, Selangor. The Clitoria plant examined (voucher specimen MFI 0176/20) was verified by botanist Dr. Mohd Firdaus Ismail and deposited in the herbarium of the Institute of Bioscience, UPM. The whole part of the plant (root, flower, and leaves) was harvested, cleaned, and placed at −80 °C prior to being freeze-dried, ground, and sieved to fine powder.

### 3.3. Extraction and Successive Fractionation of CT

The extraction was performed using solid-to-liquid ratio 1:15 of each fine-powdered part of plant (root (CTR), leaves (CTL), and flower (CTF)) with 100% methanol at room temperature with ultrasonic assistance while maintaining the sonication bath temperature between 30 and 40 °C [59]. After 3 cycles of 30 min each, the extraction mixture was filtered, concentrated in vacuo, and further lyophilized to obtain roughly non-moisturized crudes. The most bioactive crude extract of methanolic CTF then underwent excessive fractionation in methanol: water system (ratio 5:2) using different solvents of increasing polarity (*n*-hexane, chloroform, ethyl acetate, butanol, and water). Each solvent fraction was evaporated under reduced pressure at 40 °C and stored at 4 °C.

### 3.4. Biological Evaluation

#### 3.4.1. Cell Culture

BV-2 cells were firstly cultured in 8 mL of DMEM medium with the 0.1% heat-inactivated FBS, supplemented with 0.01% of penicillin/streptomycin (100 U/mL of penicillin and 100 µg/mL of streptomycin) for complete growth medium (CGM) in a 25 cm^3^ cell culture flask, before being incubated under 5% CO_2_ at 37 °C. Once the flask reached 90% confluence, the medium-containing suspension cells were firstly discarded, while the remaining adherent cells were then trypsinized with a 10× trypsin–EDTA solution (500 µL), incubated for approximately 3–4 min, and gently tapped for 1 min to dislodge the cells. As the attached cells were dispersed (checked under a microscope), CGM (3 mL) was added to inactivate the trypsin, and the cell suspensions were centrifuged at 1000 rpm/5 min. Following that, the supernatant was discarded, and the cell pellet was re-suspended in CGM (1 mL) and sub-cultured into a new 25 cm^3^ flask containing 8 mL of CGM at the sub-cultivation ratio of 1:4 (attached cell ratio on volume of flask surface area), before being further incubated in 5% CO_2_ at 37 °C. Meanwhile, SK-N-SH cells were cultured following the same procedures as BV-2 cells except that they were maintained in EMEM culture medium, supplemented with 0.1% heat inactivated FBS, and 0.01% of penicillin/streptomycin (100 U/mL of penicillin and 100 µg/mL of streptomycin) for a CGM. Cells were cultured once a week at the ratio of 1:8 (attached cell: volume of flask surface area).

#### 3.4.2. Cell Viability Determination (MTT Assay)

The 2 × 10^4^ cells of BV-2 and 4 × 10^4^ SK-N-SH cells in 180 µL of CGM were seeded in each well of 96-well plates and were allowed to be incubated in 5% CO_2_ at 37 °C for 24 h for cell attachment purpose. The extracts (CTR, CTL, and CTF) and the most bioactive fractions of CTF (*n*-hexane, chloroform, ethyl acetate, butanol, and water) were dissolved in DMSO and then serial diluted using CGM with concentrations of 0.1 µg/mL, 1 µg/mL, 10 µg/mL, and 100 µg/mL. A total of 20 µL of each diluted extract and fraction was added into wells in four replicates and incubated for 24 h at 37 °C. For assay termination, 50 µL of 3-(4,5-dimethylthiazol-2-yl)-2,5-diphenyltetrazolium bromide reagent (MTT; 2 mg/mL in PBS) was added into each well and incubated for 4 h in 5% CO_2_ at 37 °C. Then, the supernatant was removed from each well, and 100 µL DMSO was added into each well to dissolve the formazan crystal formed. The absorbance was read at 550 nm using a VersaMax (Molecular Device Analytical Technologies LLC, San Jose, CA, USA) microplate reader [60].

#### 3.4.3. Nitric Oxide Inhibitory Assay (Griess Assay)

The 2 × 10^4^ cells of BV-2 were seeded in a 96-well plate and incubated for 24 h to allow the attachment of cells. The cells were pretreated with extracts and fractions at different concentrations (0.1 µg/mL, 1 µg/mL, 10 µg/mL, 100 µg/mL) and were incubated for 24 h. The cells were then stimulated with 1 µg/mL of LPS (*Escherichia coli*, serotype 0111:B4) and further incubated for 24 h. The nitric oxide (NO) level was then determined by a Griess assay, where 50 µL supernatant in each well was transferred into a new 96-well plate and mixed with 50 µL Griess reagent (1% sulphanilamide and 0.1% *N*-(1-naphthyl) ethylenediaminedihydrochloride in 5% phosphoric acid) at room temperature. The absorbance was measured at 540 nm using a VersaMax (Molecular Device Analytical Technologies LLC, San Jose, CA, USA) microplate reader. The concentration of nitrites was quantified using an equation generated from a standard curve constructed using serial dilutions of sodium nitrite as a standard. Meanwhile, the percentage of inhibition was calculated based on ability of extract/fraction to inhibit nitrite production below the levels produced by BV-2 cells [60].

#### 3.4.4. Statistical Analysis

The MTT and NO-inhibitory results of CT extracts and fractions on both LPS-activated BV-2 and SK-N-SH cells were expressed as the mean of 3 replicates ± standard deviation (SD). Data for the various parameters were subjected to one-way analysis of variance (ANOVA) using the software Graphpad Prism v8.0.2.263 (Graphpad Software, San Diego, CA, USA) to determine statistical significance. The values were considered significant when *p* < 0.05.

### 3.5. Ultra-High-Performance Liquid Chromatography–Tandem Mass Spectrometry (UHPLC–MS/MS) Analysis

The crude extracts and fractions were submitted for metabolites profiling determination by ultra-high-performance liquid chromatography–tandem mass spectrometry (UHPLC–MS/MS). For sample preparation, 5 mg of the extract/fraction was dissolved in 1 mL of LC–MS grade methanol and filtered using a 0.22 µm hydrophobic PTFE membrane. The analysis was performed on a Dionex UHPLC-PDA system interfaced to a Q-Exactive Plus Mass Spectrometer (Thermo Scientific, San Jose, CA, USA) using a heated electrospray ionization (HESI-II) source. The LC condition for both instruments was standardized as follow: Waters BEH C_18_ UPLC column (150 mm × 2.1 mm, 1.7 µM); mobile phase: (A) water with 0.1% formic acid, (B) acetonitrile with 0.1% formic acid. The gradient elution of solvent system is 5–100% B over 30 min and isocratic at 100% B for 5 min; flow rate: 400 µL/min; and injection volume: 2 µL. Meanwhile, the optimized HESI-II parameters were as follows: spray volt-pressure −3.6 kV; sheath gas flow rate at 50 units; auxiliary gas flow rate: 8 units; equipment temperature: 40 °C; capillary temperature: 300 °C; scan range: 150–1200 *m/z*; and collision-induced dissociation (CID) energy was adjusted to 30%. The data were collected and processed using Thermo Xcalibur Qual Browser software 4.0 [61].

### 3.6. Generation of Molecular Networks

The Thermo RAW files obtained from the UHPLC–MS/MS analysis were firstly converted into .mzXML format using MSConvert GUI of ProteoWizard (http://proteowizard.sourceforge.net/tools.html, accessed on 15 June 2021). Then, the .mzXML data were uploaded to the Global Natural Product Social Molecular Networking (GNPS) website (http://gnps.ucsd.edu, accessed on 16 June 2021) to create a molecular network (MN) for the most bioactive CTF, as well as for the ethyl acetate fractions of CTF. The spectral network was functioned as window-filtered by choosing only the top 6 peaks in the ±50 Da window throughout the program. The consensus spectra were created with a parent ion mass tolerance of 0.02 Da and fragment ion mass tolerance of 0.02 Da. Following that, the consensus spectra with fewer than two spectra were removed. A network was then created with its edges having a cosine score above 0.7 and more than six matched peaks. Further edges between two nodes were kept in the network if and only if each of the nodes appeared in each other’s respective top 10 most similar nodes. Then, the spectral in the network was searched against the spectral GNPS library. The spectral was matched with the spectral library if it had at least 6 matched fragment ions. The MN created was then further annotated against in silico spectral libraries: MassBank of North America (MONA), Metabolomic Workbench Database, and MassBank Europe and was restricted at various taxonomical levels. Spectral matching parameters were set as following: TOLERENCE = 0.005, SCORE_TRESHOLD = 0.2, TOP_K_RESULTS = 5. The MN was then visualized using Cytoscape 3.7.2 (http://www.cytoscape.org, accessed on 16 June 2021). The blank sample (100% methanol) was included in the spectral network to avoid misinterpretation of HPLC contaminants and noises [61].

### 3.7. In Silico Molecular Docking Studies

Molecular docking was performed to study the intermolecular interaction and binding mode of the major, annotated metabolites **1**, **7**, and **10** against the iNOS enzyme (PDB ID: 3E6T) [62]. This enzyme consists of co-crystallized ligands, HEM901 (protoporphyrin IX Fe[Heme]), H4B902 (5,6,7,8-tetrahydrobiopterin), and 1A2905 (5-(4′-amino-1′-ethyl-5′,8′-difluoro-1’h-spiro[piperidine-4,2′-quinazoline]-1-ylcarbonyl)picolinonitrile) with water molecules. Since 1A2905 plays the role of an inhibitor, the environment in which this 1A2905 is located is an active site. Therefore, 1A2905 was extracted from the iNOS enzyme and used to validate the docking protocol. In addition, the PDB file of the iNOS enzyme was submitted to the Computed Atlas of Surface Topography of proteins (CASTp) 3.0 server [63] to predict the active site residues.

Prior to docking simulation, the ligands, water, and chain B were removed from the target protein using Discovery Studio Visualizer (DSV) 19.1. All missing hydrogen atoms and Gasteiger charges were then added to the target protein and ligands (compounds **1**, **7**, and **10**) using Autodock Tools 1.5.6 [64]. A grid box which covered all active site residues was established. Molecular docking was then performed using AutoDock 4.2.6 software [64] with the Lamarckian genetic algorithm (LGA) approach, and the remaining parameters were set as default values. Docking results were selected based on lowest binding energy and interactions were analyzed and visualized using DSV 19.1.

### 3.8. In Vivo Toxicity in Zebrafish Embryos

All zebrafish experiments were performed according to the approved guidelines and regulations of the Institutional Animal Care and Use Committee (IACUC) of Chungnam National University.

#### 3.8.1. Zebrafish Husbandry and Embryo Collection

Embryos were obtained by natural mating of wild-type (WT) or blood-vessel-specific EGFP-fluorescent transgenic zebrafish line *Tg(kdrl:egfp)*. Adult zebrafish were obtained from the Zebrafish Center for Disease Modeling (ZCDM; Daeheon, Republic of Korea). Fish were housed in a mixed enclosure for males and females in a 2:3 ratio, with a controlled photoperiod of 14 h light: 10 h dark and an ambient temperature of 28.5 °C. They were fed four times daily, alternating between brine shrimp (Artemia, INVE aquaculture Inc, Salt Lake City, UT, USA) and commercial flake food (Gemma Micro 75 zf, skretting France, Le Pont de Pierre, France). Embryos were collected 30 min after fertilization and further incubated at 28 °C. The fertilized embryos were then selected and examined under a dissecting microscope (Leica S6E, KL 300 LED, Schott, Germany) prior to the assays’ execution.

#### 3.8.2. Acute Toxicity Testing on Zebrafish Embryos

To test the acute toxicity of CTF_EA on early embryonic development, twenty zebrafish embryos at the 4 hpf stage were placed in each well of a 24-well plate containing 1 mL of embryo medium. CTF_EA was dissolved in dimethyl sulfoxide (DMSO) to make a 50 mg/mL stock solution and serially diluted into the embryonic water at concentrations of 200 µg/mL, 100 µg/mL, and 50 µg/mL. The test solution was changed daily to maintain the freshness of the solutions. For the microscopic images, zebrafish embryos were dechorionized with forceps, anesthetized with Tricaine (Sigma-Aldrich, St. Louis, MO, USA), and embedded in 3% methylcellulose at 24 hpf, 48 hpf, and 72 hpf. Images were captured using a Leica MZ16, S6E stereomicroscope (Schott, Germany), while digital images were captured using a Leica DFC450C digital camera (Leica TL5000 Ergo transmitted light base) and processed using Leica Application Suite (Leica, Wetzlar, Germany) [65].

#### 3.8.3. Tail-Coiling Movements in Zebrafish Embryos

The first locomotor activity in zebrafish starts with spontaneous tail-coiling movements. This simple behavioral pattern involves a side-to-side movement of the tail mediated by a central pattern generator of motoneurons and interneurons in the spinal cord. Spontaneous tail-coiling movements were studied in embryos aged 24 to 26 hpf, and only embryos that did not exhibit malformations were selected. The number of tail coils was counted manually per 1 min under a standard dissecting microscope. Embryos were habituated for 2 min before tail-coiling counting was started. A complete cycle of coiling was represented by a whole-body contraction that brings the tail tip to the head and includes two alternating lateral (left–right) bends of the entire body [66].

#### 3.8.4. Evaluation of Adverse Effect on Development of Blood Vessels

The transgenic zebrafish line *Tg(kdrl:egfp)* was treated with 0.1% DMSO as control or with different concentrations of CTF_EA (100 µg/mL, 50 µg/mL, and 25 µg/mL). Treatment with CTF_EA began at the developmental stage of 10 hpf and was examined at 30 hpf when normal blood vessel formation was observed in control zebrafish. For bioimaging, treated embryos were embedded in 3% methylcellulose on a glass slide, and images of live animals were acquired using a CELENA^®^ S Digital Imaging System (Logos Biosystems, Anyang, Korea) [65].

#### 3.8.5. Evaluation of Cell Death and Apoptosis

Assessment of cell death in wild-type zebrafish larvae (WT) after treatment with CTF_EA was determined by the method of vital fluorescent dye acridine orange, which is commonly used as a marker of apoptotic cells in zebrafish. Ten zebrafish embryos were placed in the egg water containing 4 µg/mL acridine orange (Sigma) for 20 min in each assay. The live zebrafish larvae were then washed with the egg water 5 times for 5 min each, anesthetized with tricaine, and embedded in 3% methylcellulose before being examined by stereomicroscopy and fluorescence microscopy as previously described [65].

#### 3.8.6. Statistical Analysis

Statistical analyses were performed with SPSS (SPSS v. 25.0). One-way analysis of variance (ANOVA) was used to determine the effect of each treatment group on the control. Data were presented as mean ± standard error (SEM) using GraphPad Prism (Graphpad Software, USA). Data were significantly different when *p* ≤ 0.05. To ensure that these values were independent, data were analyzed per well to avoid interaction bias between embryos.

## 4. Conclusions

In summary, the ethyl acetate fraction of *C. ternatea* flower extracts (CTF _EA) exhibited significant NO suppression in a dose-dependent manner at all concentrations tested, while cell viability was maintained up to ~90% in LPS-induced microglia BV-2 and neuroblastoma SK-N-SH cell lines. In the present study, the concept of bioassay-guided molecular networks (MN) was applied as a systematic strategy to uncover and facilitate dereplication and/or annotation of bioactive molecules in a complex CTF_EA metabolome to discover the major clusters of flavonol 3-*O*-glycosides, hydrocinnamic acids, and derivatives, as well as primary metabolites as compounds responsible for their neuroprotective and anti-neuroinflammatory properties. Molecular docking studies suggested that CTF _EA preferentially targets iNOS, leading to a reduction in the production of NO. In addition, no toxic effects on normal embryonic development, blood vessel formation, and apoptosis were observed when CTF_ EA was tested for in vivo toxicity in zebrafish animal models. The overall preliminary results suggest the anti-neuroinflammatory and neuroprotective potentials of CT, thus, scientifically supporting the efficacy of this medicinal plant at both local and traditional levels. However, studies of the targeted isolation of bioactive metabolites, in-depth pharmacological efficacy, and safety in vitro and in vivo in a larger mammalian model are urgently needed to deepen our understanding of this plant before it can be developed into a promising therapeutic agent to slow down the progression of AD, particularly in the near future.

## Figures and Tables

**Table 1:** The metabolites identified in the ethyl acetate fraction of flower *Clitoria ternatea* extract (CTF_EA).

Peak No.	Putative Identification	Molecular Formula	RT (min)	Precursor Ion (*m/z*)	Ion Type	Main Fragments (*m/z*)	Wavelength (nm)	References
Cluster A: Flavonol 3-*O*-glycosides								
**1.**	Kaempferol-3-*O*-rutinoside	C_27_H_30_O_15_	5.59	593.1503	[M-H]^−^	284, 285, 255, 227, 151	208, 220, 266, 350	GNPS
**2.**	Kaempferol-3-*O*-glucoside	C_21_H_20_O_11_	6.28	447.1345	[M-H]^−^	284, 285, 255, 227, 151	218, 266, 294, 344	GNPS
**3.**	Kaempferol 3-(6″-acetyl-glucoside)	C_23_H_22_O_12_	6.80	489.1045	[M-H]^−^	285, 284, 255, 227	220, 272, 294	Metabolomics workbench
**4.**	Kaempferol 3-(6G-malonyl-neohesperidoside)	C_30_H_32_O_18_	6.16	679.1527	[M-H]^−^	635, 285, 284, 255, 227	220, 268, 298, 314, 344	Metabolomics workbench
**5.**	Kaempferol-3-*O*-α-*L*-rhamnosyl-(1->2)-*O*-*L*-rhamnoside	C_27_H_30_O_14_	7.28	577.269	[M-H]^−^	285, 284, 255, 227	220, 268, 298	Metabolomics workbench
**6.**	Kaempferol 3-*O*-(4″-*O*-acetyl)-rutinoside	C_29_H_32_O_16_	6.89	635.162	[M-H]^−^	284, 285, 255, 227, 151	220, 268, 298, 368	https://mona.fiehnlab.ucdavis.edu/ (accessed on 13 August 2021)
**7.**	Kaempferol-3-*O*-(2-rhamnosyl)-rutinoside	C_33_H_40_O_19_	5.20	739.1042	[M-H]^−^	284, 285, 255, 227, 151	198, 266, 350	GNPS
**8.**	Avicularin	C_20_H_18_O_11_	5.82	433.0780	[M-H]^−^	301, 300, 271, 255, 151	220,268,312	GNPS
**9.**	Quercetin-3-*O*-deoxyhexosyl- (1–2) pentoside	C_26_H_28_O_15_	5.70	579.1364	[M-H]^−^	301, 300, 271, 255, 151	218, 266, 350	GNPS
**10.**	Rutin	C_27_H_30_O_16_	5.40	609.1461	[M-H]^−^	301, 300, 271, 255, 151	206, 258, 354	GNPS
**11.**	Isoquercetin	C_21_H_20_O_12_	5.51	463.0887	[M-H]^−^	301, 300, 271, 255, 151	206, 266, 350	GNPS
**12.**	Quercetin 3-(2G-glucosyl-rutinoside)	C_33_H_40_O_21_	5.84	771.1786	[M-H]^−^	609, 463, 301, 300, 271, 255, 151	220, 268, 314	Pubchem
**13.**	Manghaslin	C_33_H_40_O_20_	4.72	755.2035	[M-H]^−^	301, 300, 271, 255, 151	206, 256, 354	Metabolomics workbench
Cluster B: Hydrocinnamic acids and derivatives								
**14**	3-Phenyl-2-[(2*S*,3*R*,4*S*,5*S*,6*R*)-3,4,5-trihydroxy-6-[[(*E*)-3-(4-hydroxy-phenyl)prop-2-enoyl]oxymethyl]-oxan-2-yl]oxyprop-2-enoic acid	C_24_H_24_O_10_	7.19	471.1300	[M-H]^−^	307, 163, 145, 119	220, 296, 368	GNPS
**15**	Dimer 3-phenyl-2-[(2*S*,3*R*,4*S*,5*S*,6*R*)-3,4,5-trihydroxy-6-[[(*E*)-3-(4-hydroxy-phenyl)prop-2-enoyl]oxymethyl]-oxan-2-yl]oxyprop-2-enoic acid		6.84	941.2737	[M_2_-H]^−^	779, 471, 163, 145	220, 272, 296, 368	Putative annotation
**16**	3-(benzoyloxy)-2-hydroxypropyl β-*D*-glucopyranosiduronic acid	C_16_H_20_O_10_	2.04	371.0984	[M-H]^−^	370, 304, 174, 163, 146, 119	194, 298, 368	GNPS
**17**	Feruloylquinic acid isomer	C_17_H_20_O_9_	6.23	367.1036	[M-H]^−^	303, 254, 193, 175, 160, 149, 134	218, 266, 346	GNPS
**18**	Caffeic acid *O*-glucoside	C_15_H_18_O_9_	2.24	341.0877	[M-H]^−^	179, 135	214, 292, 368	https://mona.fiehnlab.ucdavis.edu/ (accessed on 25 August 2021)
**19**	*p*-Coumaric acid 4-*O*-glucoside	C_15_H_18_O_8_	3.28	325.1843	[M-H]^−^	163, 145, 119	214, 290, 368	(1) https://mona.fiehnlab.ucdavis.edu/ (accessed on 25 August 2021) (2) Metabolomics workbench
**20**	3,5-Di-*O*-caffeoyl-4-*O*-coumaroylquinic acid	C_34_H_30_O_14_	6.78	661.1782	[M-H]^−^	205,163, 145, 119	220, 292, 368	Metabolomics workbench (accessed on 25 August 2021)
Cluster C: Glycerophospholipid								
**21**	Lysophosphatidylmyoinositol	C_27_H_53_O_12_P	21.89	599.3205	[M-H]^−^	283, 241, 152	224	GNPS
**22**	Dipalmitoylphosphatidylglycerol	C_38_H_75_O_10_P	35.31	721.3657	[M-H]^−^	255	220	GNPS
**23**	Phosphatidylinositol lyso 16:0	C_25_H_49_O_12_P	18.61	571.2889	[M-H]^−^	255, 241, 152	224	https://mona.fiehnlab.ucdavis.edu/ (accessed on 2 September 2021)
**24**	1,2-Dioctanoyl-sn-glycero-3-phospho-1*D*-myo-inositol	C_25_H_47_O_13_P	20.18	585.3047	[M-H]^−^	269, 241, 152	224	Pubchem
Cluster D: Amino acids								
**25**	*N*-Fructosyl pyroglutamate	C_11_H_17_NO_8_	0.66	290.0803	[M-H]^−^	200. 128	196, 264, 370	(1) https://mona.fiehnlab.ucdavis.edu/ (accessed on 7 September 2021)(2) Metabolomics workbench
**26**	Diglucoside pyroglutamate	C_17_H_27_NO_13_	0.72	470.1507	[M-H]^−^	128	266	Putative annotation (accessed on 7 September 2021)
**27**	Fructosylvaline	C_11_H_21_NO_7_	0.74	278.1246	[M-H]^−^	214, 128, 116	256, 266	Pubchem
**28**	Agropinic acid	C_11_H_19_NO_8_	0.86	292.8916	[M-H]^−^	274, 128	204, 260	Pubchem
Cluster E: Carbohydrates								
**29**	Sucrose	C_12_H_22_O_11_	2.24	341.0877	[M-H]^−^	179, 135	214, 292, 368	GNPS
**30**	Sucrose adduct chloride	C_12_H_22_O_11_	0.64	377.0854	[M+Cl]^−^	341, 215, 179, 89, 59	194, 266, 370	Literature
**31**	6-epi-7-Isocucurbic acid glucoside	C_18_H_30_O_8_	7.33	373.1871	[M-H]^−^	174, 119, 113, 101, 89, 71, 59	220, 268, 298, 368	Pubchem
**32**	(2*R*,3*R*,4*S*,5*S*,6*R*)-2-[(3S,4S,5R)-3,4-Dihydroxy-2,5-bis(hydroxymethyl)-oxolan-2-yl]oxy-3,4,5-trihydroxy-6-(hydroxymethyl)oxan-2-yl] 3-methylbutanoate	C_17_H_30_O_13_	5.62	441.1745	[M-H]^−^	330, 139, 119, 113, 101, 89, 71, 59	208,266, 350	Pubchem
**33**	Methyl 2-[(1*R*)-2-[(*Z*)-pent-2-enyl]-3-[(2*R*,3*R*,4*S*,5*S*,6*R*)-3,4,5-trihydroxy-6-(hydroxymethyl)oxan-2-yl]oxy-cyclopentyl]acetate	C_19_H_32_O_8_	3.71	387.1152	[M-H]^−^	352, 274, 163, 113, 101, 89, 71, 59	216, 272, 298	Pubchem
Cluster F: Mono-methoxylflavonol 3-*O*-glycoside								
**34**	Isorhamnetin-3-galactoside-6’’-rhamnoside	C_28_H_32_O_16_	5.70	623.1410	[M-H]^−^	315, 314, 299, 271, 151	218, 266, 350	GNPS
**35**	Rhamnetin-3-*O* -gentiobioside	C_28_H_32_O_17_	5.03	639.2764	[M-H]^−^	315, 314, 299, 271, 255, 165, 121	204, 258, 354	GNPS
**36**	3-((6-(((3,5-Dihydroxy-6-methyl-4-((3,4,5-trihydroxy-6-methyl-tetrahydro-2*H*-pyran-2-yl)oxy)-tetrahydro-2*H*-pyran-2-yl)oxy)-methyl)-3,4,5-trihydroxytetrahydro-2*H*-pyran-2-yl)oxy)-5,7-dihydroxy-2-(4-hydroxy-3-methoxyphenyl)-4*H*-chromen-4-one	C_34_H_42_O_20_	5.32	769.2203	[M-H]^−^	605, 314, 299, 271, 151	206, 258, 354	Putative annotation
Cluster G: Saccharolipid								
**37**	1-*O*-[(2*E*)-6-[[3,4-bis-*O*-[(2*E*)-6-hydroxy-2,6-dimethyl-1-oxo-2,7-octadien-1-yl]- β-*D*-glucopyranosyl]-oxy]-2,6-dimethyl-1-oxo-2,7-octadien-1-yl] β-*D*-Glucopyranose,	C_42_H_64_O_17_	4.69	885.1616	[M+HCOO-]^−^	839, 793, 491, 399, 356, 303	206, 256, 354	https://mona.fiehnlab.ucdavis.edu/ (accessed on 25 September 2021)

**Table 2:** Comparison of binding energy between compound **1, 7,** and **10** with target enzyme inhibitors.

Enzyme	Binding Energy (kcal/mol)			
	Co-Crystallized Ligand	Compound 1	Compound 7	Compound 10
P38	−8.85	−7.82	−2.29	−6.79
ERK-2	−7.55	−7.08	−6.04	−5.78
iNOS	−7.64	−10.11	−8.78	−8.33
JNK	−9.24	−7.95	−7.79	−6.69
COX-2	−10.55	−4.98	−4.59	−3.47

**Table 3:** Binding energy and intermolecular interactions of compound **1**, **7**, and **10** against iNOS enzyme.

Compound	Binding Energy (kcal/mol)	Interactions			
		Hydrogen Bond	Hydrophobic		
			π–Alkyl	π–Sigma	π–π Stacked
**1**	−10.11	Thr184, 3 Arg193, Cys194, Gly196, Pro344, Asn364	2 Cys194, Leu203	Ala191	2 Trp188, 2 Phe363
**7**	−8.78	Arg193, Cys194, Trp366, 3 Glu371, Asp376	Ala191, Arg193	Val346	
**10**	−8.33	Arg193, Pro344, Ala345, Asn364, Tyr367, Tyr483	Ala191, Arg193, Cys194, Met149	Ala191, Cys194	Trp188, 2 Phe363

## Data Availability

The data presented in this study are available within the article and Supplementary Materials.

## Supplementary Materials

The following supporting information can be downloaded at: https://www.mdpi.com/article/10.3390/ph15040467/s1, Figure S1: Full visualization of the molecular network for the ethyl acetate fraction of flower *C. ternatea* (CTF_EA) generated from the (A) positive and (B) negative ion modes of MS/MS spectral data; Figure S2: Glycerophospholipid (Cluster C in negative mode) from the full molecular network (MN) of ethyl acetate fraction of flower *C. ternatea* (CTF_EA) extract. The metabolites in red boxed were annotated based on the GNPS library matching; Figure S3: Major fragment ions generated through the fragmentation of glycerophospholipids; Figure S4: Amino acid (Cluster D in negative mode) from the full molecular network (MN) of ethyl acetate fraction of flower *C. ternatea* (CTF_EA) extract; Figure S5: Major fragment ions from the fragmentation of (A) pyroglutamate- and (B) glutamine-based amino acids; Figure S6: Carbohydrates (Cluster E in negative mode) from the full molecular network (MN) of ethyl acetate fraction of flower *C. ternatea* (CTF_EA) extract. The metabolite in red box was annotated based on the GNPS library matching; Figure S7: Proposed fragmentation of deprotonated glucoses; Figure S8: Saccharolipids (Cluster G in negative mode) from the full molecular network (MN) of ethyl acetate fraction of flower *C. ternatea* (CTF_EA) extract; Figure S9: Proposed fragmentation in MS/MS spectra of saccharolipid; Figure S10: The validation of accuracy and performance of AutoDock 4.2.6. The re-docked 1A2905 (pink) and the native 1A2905 (blue) showed an RMSD of 0.4662 Å.
