# Supplementary material for: Mapping Molecular Networks within Clitoria ternatea Linn. against LPS-Induced Neuroinflammation in Microglial Cells, with Molecular Docking and In Vivo Toxicity Assessment in Zebrafish
Source: Pharmaceuticals (Basel). 2022 Apr 12;15(4):467. doi: 10.3390/ph15040467 (PMC9032563; doi:10.3390/ph15040467)
Supplement: Supplementary file 1 [file pharmaceuticals-15-00467-s001.zip › pharmaceuticals-1651378-supplementary.pdf]

SUPPLEMENTARY MATERIAL

Mapping Molecular Networks within *Clitoria ternatea* Linn. Against LPS-Induced Neuroinflammation in Microglial Cells, with Molecular Docking and *In Vivo* Toxicity Assessment in Zebrafish

Nurul Farah Adni Mat Zian [1,†], Puspanjali Swain [2,†], Siti Munirah Mohd Faudzi [1,3,*], Norzalina Zakaria [3], Wan Norhamidah Wan Ibrahim [1,4], Noraini Abu Bakar [4], Khozirah Shaari [1], Johnson Stanslas [5], Tae-Ik Choi [2] and Cheol-Hee Kim [2,*]

[1] Natural Medicines and Product Research Laboratory, Institute of Bioscience, Universiti Putra Malaysia, Serdang 43400, Selangor, Malaysia;
[2] Department of Biology, Chungnam National University, 99 Daehak-ro, Yuseong-gu, Daejeon 34134, Korea;
[3] Department of Chemistry, Faculty of Science, Universiti Putra Malaysia, Serdang 43400, Selangor, Malaysia;
[4] Department of Biology, Faculty of Science, Universiti Putra Malaysia, Serdang 43400, Selangor, Malaysia;
[5] Department of Medicine, Faculty of Medicine & Health Sciences, Universiti Putra Malaysia, 43400 Serdang, Selangor, Malaysia;

* Correspondence: sitimunirah@upm.edu.my (S.M.M.F.); zebrakim@cnu.ac.kr (C.-H.K.)
† These authors contributed equally.

1. Molecular Network

The chemical space of ethyl acetate fraction of *Clitoria ternatea* flower extracts (denoted as CTF_EA) was further analysed in detail by mapping its structurally related compounds into MN. To achieve this, the acquired large datasets of MS/MS fragmentation spectra were organized as MNs by using the classical workflow available on the GNPS experimental workflow (http://gnps.ucsd.edu). The MNs generated from the positive and negative modes' mass spectral data of the CTF_EA are shown in Figure S1. The following subsections discuss the annotation of the metabolites of selected clusters C, D, E, and G (not discussed in the main manuscript) as mapped in the network, along with their respective fragmentation pathways.

1.1 Glycerophospholipid (Cluster C)

The automated GNPS spectral library matching allowed dereplication of several glycerols in cluster C (Figure S2). The nodes with precursor ions at *m/z* 599.3205 $[M-H]^-$ and *m/z* 721.3657 $[M-H]^-$ were annotated as lysophosphatidylmyoinositol (**21**) and dipalmitoyl-phosphatidylglycerol (**22**), respectively. Meanwhile, examination of the MS/MS spectral data of the neighboring nodes with precursor ions at *m/z* 571.2889 $[M-H]^-$ and *m/z* 585.3047 $[M-H]^-$ led to the identification of the corresponding phosphatidylinositol lyso 16:0 (**23**) and 1,2-dioctanoyl-sn-glycero-3-phospho-1D-myo-inositol (**24**). In particular, the fragmentation pattern of the skeletal chain of glycerophospholipids, **21**, **23** and **24**, reveals the natural loss of fatty acids from the glycerol units, as observed at *m/z* 283, *m/z* 255 and *m/z* 269, respectively (Hsu & Turk, 2009).

In addition, fragment ions at *m*/*z* 241 provided further important information on the number of saturated carbons within the parent chain of these identified compounds. While dipalmitoylphosphatidylglycerol (**22**) had the fragment of fatty acid at *m*/*z* 255. All metabolites recorded a fragment of 152Da, which is a significant structural feature of glycerolphosphate (Figure S3).

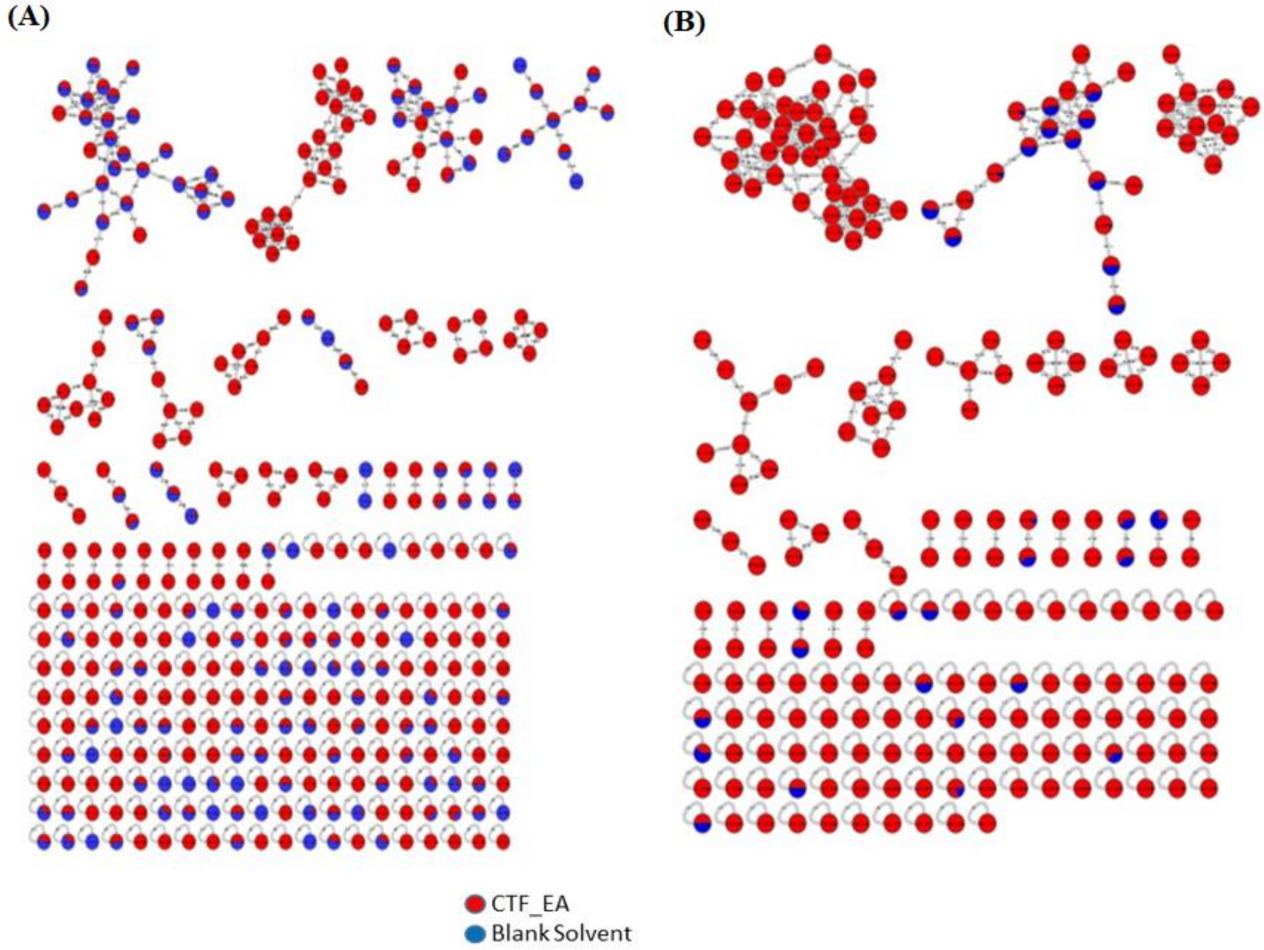

Figure S1. Full visualization of the molecular network for the ethyl acetate fraction of flower *C. ternatea* (CTF_EA) generated from the (**A**) positive and (**B**) negative ion modes of MS/MS spectral data.

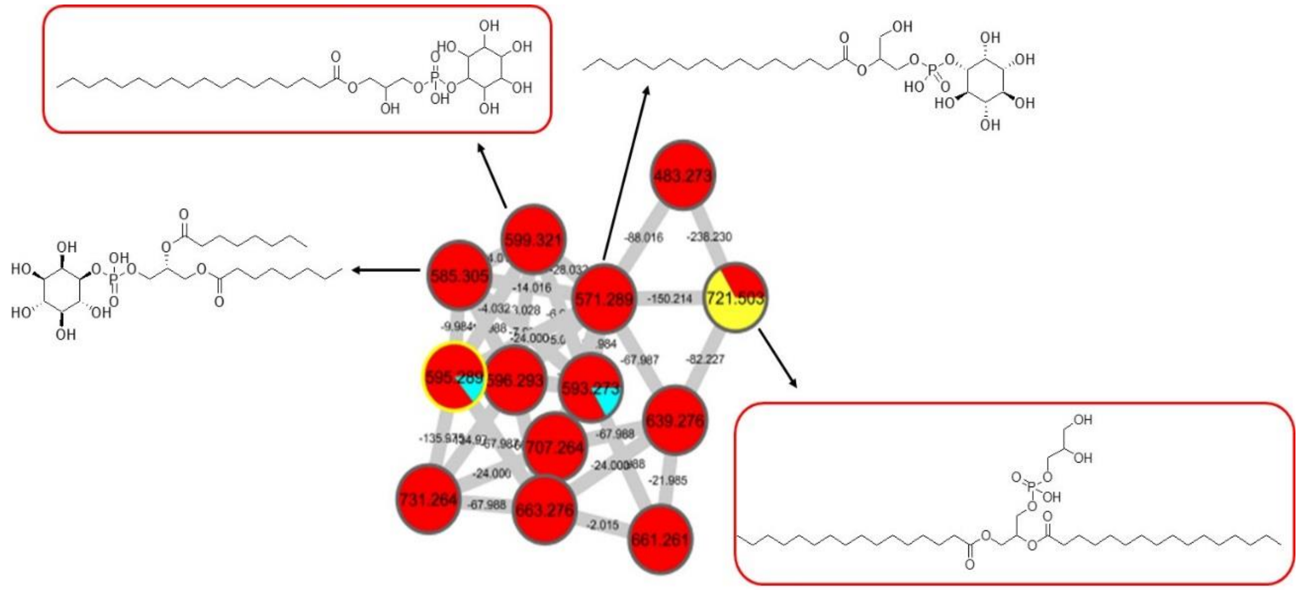

Figure S2. Glycerophospholipid (Cluster C in negative mode) from the full molecular network (MN) of ethyl acetate fraction of flower *C. ternatea* (CTF_EA) extract. The metabolites in red boxed were annotated based on the GNPS library matching.

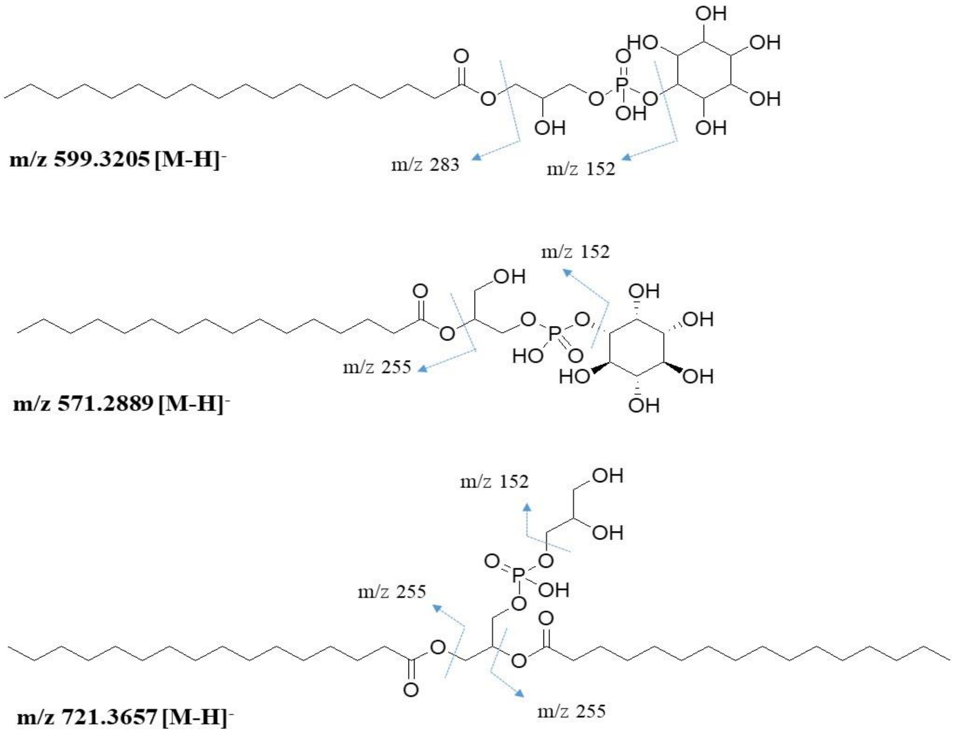

Figure S3. Major fragment ions generated through the fragmentation of glycerophospholipids.

1.2 Amino acids (Cluster D)

Visual inspection of the primary metabolites in cluster D (Figure S4) as pyroglutamate- and glutamine-based amino acid was based on the Pubchem, Massbank and Metabolomics Workbench online databases. A deprotonated molecular ion at *m/z* 290.0803 [M-H]$^-$ was identified as *N*-fructosyl pyroglutamate (**25**), showing fragment ions of pyroglutamate at *m/z* 128 for [(M-H)-sugar unit]$^-$ and *m/z* 200 for [(M-H)-$C_3H_6O_3$]. The node with *m/z* 470.151 [M-H]$^-$ was next putatively annotated as a lactulose pyroglutamate (**26**). Based on the mass spectrum, the base peak of *m/z* 128 was shown to be pyroglutamate, which was due to the cleavage of the dissacharide molecule [(M-H)-sugar unit-H_2O] [1, 2]. It is worth noting that oxidation of the C–N bond linking the carbon of the fructosyl moiety and the nitrogen of the amino group of the fructosylamino acids leads to an unstable Schiff base intermediate, which is hydrolysed to form glucosone and an amino acid [3]. Concurrently, the deprotonated *m/z* 292.8916 [M-H]$^-$ was assigned as agropinic acid (**28**) and its MS/MS spectral data showed a base peak at *m/z* 274 [(M-H)-H_2O)]$^-$ and another fragment at *m/z* 128 (fructosylamino acid) after the loss of sugar moiety. The proposed fragmentation mechanism of **25**, **26** and **28** was further validated based on the similarity of the spectrum with the Massbank library. Meanwhile, for the node containing the precursor ion [M-H]$^-$ at *m/z* 278.1246, the identity of the metabolite was assigned as fructosylvaline (**27**) based on the characteristic fragment ion of the *N*-terminal glutamine at *m/z* 116 for [(M-H)-sugar unit]$^-$. The mechanism showing the loss of pyroglutamate and glutamine structural unit is shown in Figure S5.

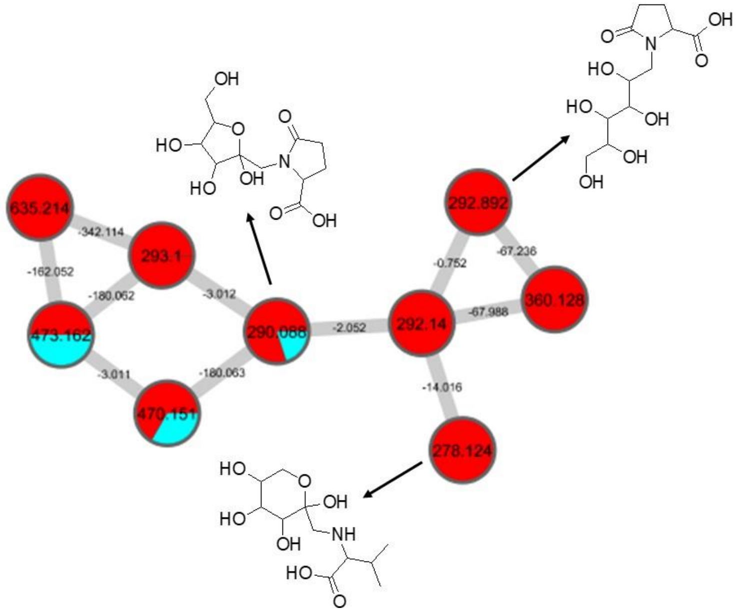

Figure S4. Amino acid (Cluster D in negative mode) from the full molecular network (MN) of ethyl acetate fraction of flower *C. ternatea* (CTF_EA) extract.

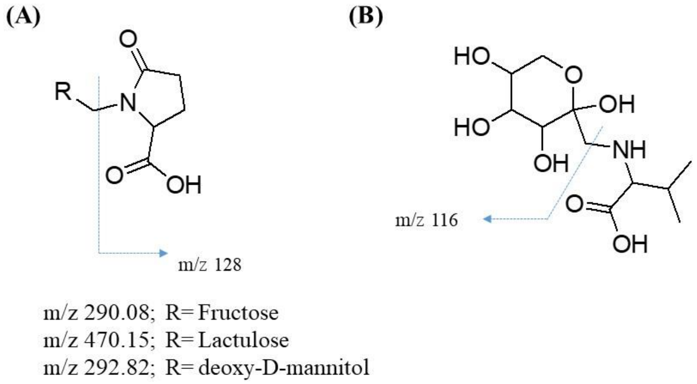

Figure S5. Major fragment ions from the fragmentation of (A) pyroglutamate- and (B) glutamine-based amino acids.

1.3 Carbohydrates (Cluster E)

Analysis of the MS/MS spectrum of this cluster E (Figure S6) shows that it belongs to the carbohydrate groups. Based on GNPS library matching, *m/z* 341.0877 [M-H]$^-$ was assigned to sucrose as it shows the loss of a hexose unit (162 Da) at *m/z* 179, indicating glycosidic cleavage at C1, while the peak at *m/z* 161 correspond to the loss of water [(M-H)-hexose-H_2O)]$^-$ [4]. Further analysis of the MS/MS spectral data revealed that nodes with *m/z* 441.75 [M-H]$^-$, *m/z* 387.1152 [M-H]$^-$ and *m/z* 373.187 [M-H]$^-$ were belong to the corresponding(2*R*,3*R*,4*S*,5*S*,6*R*)-2-[(3*S*,4*S*,5*R*)-3,4-dihydroxy-2,5-bis(hydroxymethyl)-oxolan-2-yl]oxy-3,4,5-trihydroxy-6-(hydroxymethyl)oxan-2-yl]-3-methyl-butanoate (**32**), methyl-2-[(1*R*)-2-[(*Z*)-pent-2-enyl]-3-[(2*R*,3*R*,4*S*,5*S*,6*R*)-3,4,5-trihydroxy-6-(hydroxymethyl)oxan-2-yl]oxycyclopentyl]acetate (**33**) and 6-epi-7-isocucurbic acid glucoside (**31**). All the metabolites exhibited *m/z* 59 as the base peak, followed by the fragments of *m/z* 71, *m/z* 89 and *m/z* 101 confirming that the metabolites belong to glucosides-class of compounds. The cross-ring cleavages of the deprotonated glucose are shown in Figure S7. In a meantime, *m/z* 377.085 [M-H]$^-$ was identified as the chloride adduct of sucrose (**30**) by comparison with the literature, in which the fragment ion at *m/z* 179 indicating the loss of a hexose moiety, suggesting glycosidic cleavage.

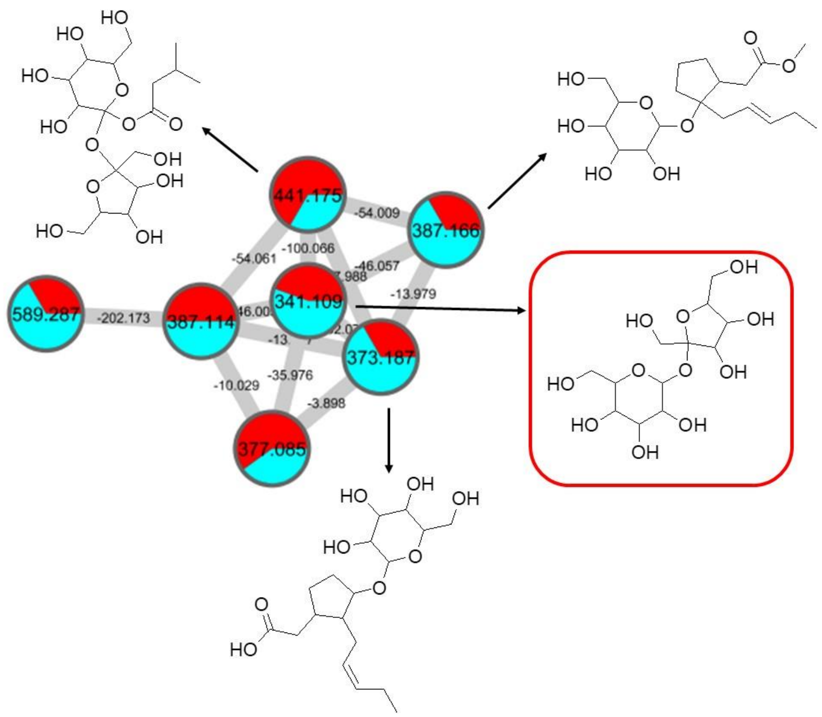

Figure S6: Carbohydrates (Cluster E in negative mode) from the full molecular network (MN) of ethyl acetate fraction of flower *C. ternatea* (CTF_EA) extract. The metabolite in red box was annotated based on the GNPS library matching.

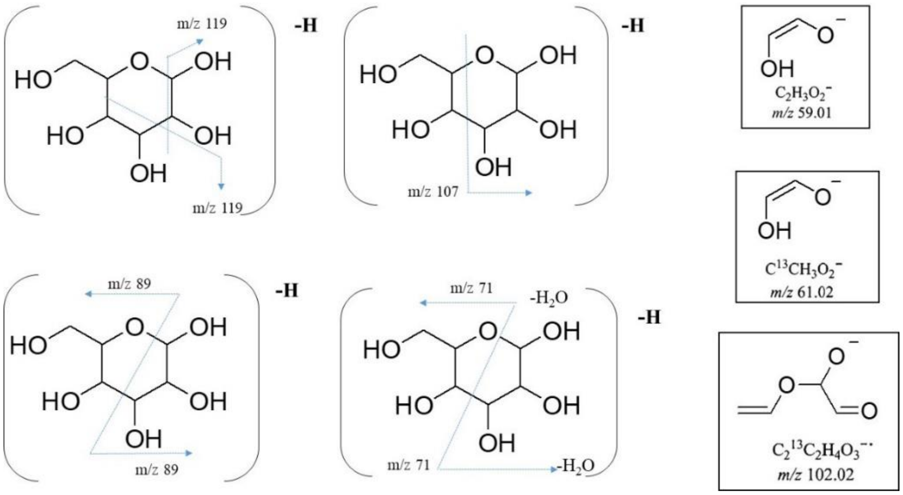

Figure S7. Proposed fragmentation of deprotonated glucoses.

1.4 Saccharolipid (Cluster G)

Massbank spectral library matching allowed the assignment of saccharolipid in cluster G (Figure S8). The precursor ion *m/z* 885.162 [(M-H)-HCOO$^-$]$^-$ contains a formic acid adduct and was identified as 1-*O*-[(2*E*)-6-[[3,4-bis-*O*-[(2*E*)-6-hydroxy-2,6-dimethyl-1-oxo-2,7-octadien-1-yl]-β-*D*-gluco-pyranosyl]-oxy]-2,6-dimethyl-1-oxo-2,7-octadien-1-yl] β-*D*-glucopyranose (**37**). The fragment ion at *m/z* 793 due to the loss of water and alkene [M-C_2H_4-H_2O]$^-$, *m/z* 491 [M-$C_{16}H_{26}O_7$-H_2O]$^-$ due to the loss of water and sugar moiety and fatty acids and *m/z* 399 as the base peak with the loss of water and sugar associated with 2 units of fatty acids [M-$C_{23}H_{34}O_7$-H_2O]$^-$ have been confirmed using the generated fragmentation from mass frontier (Figure S9).

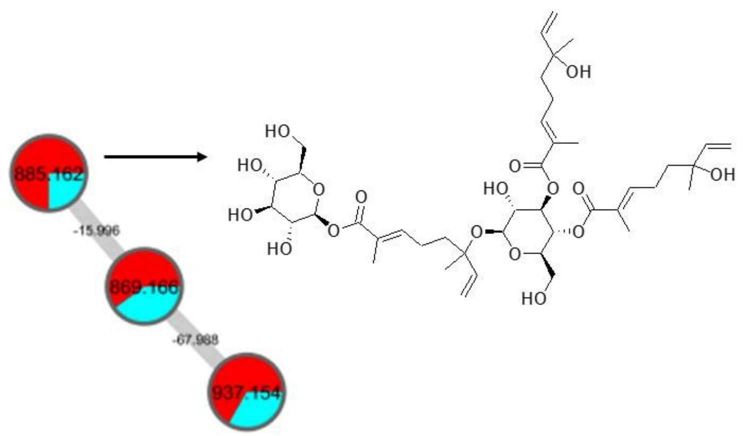

Figure S8. Saccharolipids (Cluster G in negative mode) from the full molecular network (MN) of ethyl acetate fraction of flower *C. ternatea* (CTF_EA) extract.

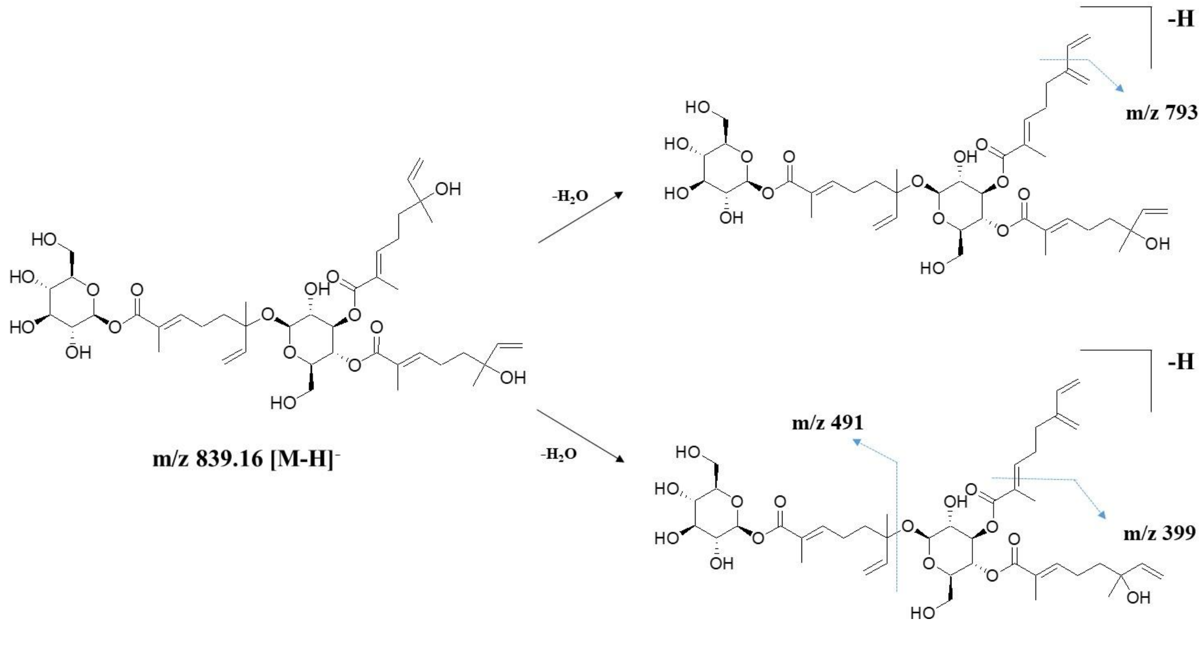

Figure S9. Proposed fragmentation in MS/MS spectra of saccharolipid.

2. Molecular docking

To validate the accuracy and reliability of the AutoDock 4.2.6 docking protocol for the present purpose, the co-crystallized 1A2905 was re-docked in the binding pocket of the iNOS enzyme obtained from the CASTp 3.0 server, and the re-docked position was compared with the position of the crystal structure by calculating the root mean square deviation (RMSD). This is a typical method to compare the structural similarity between two superimposed structures (see Figure S10), where the successful scoring function is the one with the RMSD value of ≤2.0 Å [5]. In this study, the RMSD value of the re-docked 1A2905 from the crystal structure was 0.4662 Å, indicating our docking methods are valid for the given structures and AutoDock 4.2.6.

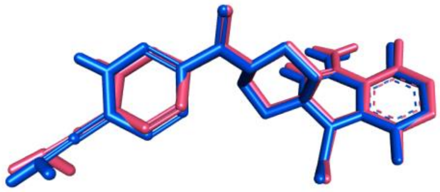

Figure S10. The validation of accuracy and performance of AutoDock 4.2.6. The re-docked 1A2905 (pink) and the native 1A2905 (blue) showed an RMSD of 0.4662 Å

References:

1. Tong, H.; Bell, D.; Tabei, K.; Siegel, M.M. Automated data massaging, interpretation, and e-mailing modules for high throughput open access mass spectrometry. *J. Am. Soc. Mass Spectrom.* **1999**. 10, 1174–1187.
2. Fisher, M.F.; Mylne, J.S. Sequencing Orbitides by Acid-Mediated Ring Cleavage Followed by Tandem Mass Spectrometry. *J. Proteome Res.* **2019**. 18, 4065–4071.
3. Ferri, S.; Kim, S.; Tsugawa, W.; Sode, K. (2009). Review of fructosyl amino acid oxidase engineering research: A glimpse into the future of hemoglobin A1c biosensing. *J. Diabetes Sci. Technol.* **2009**. 3, 585–592.
4. Calvano, C.D.; Cataldi, T.R.I.; Kögel, J.F.; Monopoli, A.; Palmisano, F.; Sundermeyer, J. Structural Characterization of Neutral Saccharides by Negative Ion MALDI Mass Spectrometry Using a Superbasic Proton Sponge as Deprotonating Matrix. *J. Am. Soc. Mass Spectrom.* **2017**. 28, 1666–1675.
5. Yusuf, D.; Davis, A.M.; Kleywegt, G.J.; Schmitt, S. An alternative method for the evaluation of docking performance: RSR vs RMSD. *J. Chem. Inf. Model*. **2008**. 48, 1411–1422.
